# The prevalence of occult leiomyosarcoma at surgery for presumed uterine fibroids: a meta-analysis

**DOI:** 10.1007/s10397-015-0894-4

**Published:** 2015-05-19

**Authors:** Elizabeth A. Pritts, David J. Vanness, Jonathan S. Berek, William Parker, Ronald Feinberg, Jacqueline Feinberg, David L. Olive

**Affiliations:** Wisconsin Fertility Institute, Middleton, WI USA; University of Wisconsin School of Medicine and Public Health, Madison, WI USA; Stanford University School of Medicine, Stanford, CA USA; University of California, Los Angeles, Los Angeles, CA USA; Reproductive Associates of Delaware, Newark, DE USA

**Keywords:** Leiomyosarcoma, Fibroids, Surgery, Incidental malignancy, Prevalence

## Abstract

There is a concern regarding the risk of occult leiomyosarcomas found at surgery for presumed benign fibroids. We sought to produce a comprehensive review of published data addressing this issue and provide high-quality prevalence estimates for clinical practice and future research. A comprehensive literature search using the PubMed/MEDLINE database and the Cochrane Library was performed. Inclusion criteria were human studies, peer-reviewed, with original data, involving cases for surgery in which fibroid-related indications were the primary reason for surgery, and histopathology was provided. Candidate studies (4864) were found; 3844 were excluded after review of the abstract. The remaining 1020 manuscripts were reviewed in their entirety, and 133 were included in the Bayesian binomial random effect meta-analysis. The estimated rate of leiomyosarcoma was 0.51 per 1000 procedures (95 % credible interval (CrI) 0.16–0.98) or approximately 1 in 2000. Restricting the meta-analysis to the 64 prospective studies resulted in a substantially lower estimate of 0.12 leiomyosarcomas per 1000 procedures (95 % CrI <0.01–0.75) or approximately 1 leiomyosarcoma per 8300 surgeries. Results suggest that the prevalence of occult leiomyosarcomas at surgery for presumed uterine fibroids is much less frequent than previously estimated. This rate should be incorporated into both clinical practice and future research.

**Electronic supplementary material:**

The online version of this article (doi:10.1007/s10397-015-0894-4) contains supplementary material, which is available to authorized users.

## Introduction

Uterine fibroids, also known as leiomyomas or myomas, are a significant gynecologic problem, affecting 70–80 % of all women during their reproductive years. These tumors are often symptomatic, producing complaints of abnormal bleeding, pain, and infertility in many of those afflicted. The disease represents a large economic burden for the health care system and significantly affects the quality of life of many with these tumors [1].

Two primary procedures have been utilized over the last century to treat myomas: the hysterectomy, for those who do not wish to retain their uterus, and myomectomy for those who wish to maintain uterine structure and function, often for future reproduction. Traditionally, these procedures were performed via a large abdominal incision (laparotomy), often required by the large size of the fibroid uterus.

Less invasive surgical approaches have been advocated and performed for many years, although with much less frequency than laparotomy. The challenge for surgeons performing these less invasive operations is the usual requirement to remove large amounts of tissue through small apertures.

Manual morcellation via scalpel or other devices has been available for decades, allowing the completion of hysterectomies (and even myomectomies) involving quite large specimens through a vaginal or mini-laparotomy route. The advent of minimally invasive surgery (MIS) utilizing endoscopy initially provided a resurgence in morcellation. As MIS skills improved among surgeons and as equipment improved in concert with the enhanced surgical skills being developed, endoscopic procedures for both hysterectomy and myomectomy increased in number and popularity. A key innovation allowing the performance of these procedures endoscopically was the development and utilization of the electromechanical (or “power”) morcellator.

The US Food and Drug Administration (FDA) first approved an electromechanical morcellation device in 1995, and a number now exist in the market. However, recently, the FDA issued a statement discouraging the use of such devices, citing safety concerns, chief among these being the inadvertent dissemination of occult uterine cancer in patients undergoing hysterectomy and myomectomy for presumed benign leiomyomata [2]. Their stated prevalence for unsuspected uterine sarcoma, based upon their review of the medical literature, was 1 in 352 for any sarcoma and 1 in 498 for leiomyosarcoma.

We and others were concerned that the FDA figures might not be the product of a comprehensive and systematic review. In response, our group decided to further investigate the prevalence of uterine leiomyosarcoma among women undergoing surgery for presumed fibroids with a thorough review of published studies of myomectomy or hysterectomy performed for the indication of symptomatic fibroids that included histopathologic analysis of all tissue removed.

### Sources

A literature search was initially performed using the PubMed/MEDLINE database and the Cochrane Library. The search was performed for all manuscripts published after 1960 and all languages using the search terms “myoma,” “leiomyoma,” “fibroid,” “hysterectomy,” “incidental malignancy,” “myomectomy,” “neoplasm,” “leiomyosarcoma,” “incidence,” “pathology,” “histopathology,” “morcellation,” and “complication.” These terms were used alone and in combination. All references found were evaluated for the inclusion and exclusion criteria listed below and their bibliographies hand-searched for other potentially relevant publications. One author (EAP) conducted a preliminary review; all papers deemed to meet inclusion and exclusion criteria were then reviewed by at least one other author for categorization (RF, JF, DLO). If a disagreement was found between reviewers, a conference involving multiple reviewers was used to reach a decision.

### Study selection

Inclusion criteria encompassed publications involving humans that were peer-reviewed. All publications were required to contain original data. Papers were included if they involved cases for surgery (hysterectomy or myomectomy) in which fibroid-related indications were the primary reason for surgery. If this was the exclusive focus of the manuscript, then all cases in the publication were extracted. If, however, there were multiple indications for surgery, only those cases with a fibroid-related primary indication were extracted and included in the analysis. To avoid case reports, a minimum of five subjects from an individual study was necessary for inclusion in this review.

Only those manuscripts in which the postoperative histopathologic findings were provided for all extracted patients were included in the analysis. Manuscripts stating “all specimens were sent to pathology” without final reports were deemed inadequate for inclusion. If the histopathologic description of a leiomyosarcoma in any study was inconsistent with the current World Health Organization (WHO) diagnostic criteria, we noted this, but included it as a leiomyosarcoma in our evaluable data [3] (see below).

Studies that initially searched their databases for a pathologic diagnosis of fibroids, then worked backward to uncover the primary indications for surgery, were excluded. Similarly, all prospective analyses that a priori excluded any patient with malignancy were excluded from the review. All letters to the editor, abstracts, and all other non-peer-reviewed publications of data were omitted. In many cases, we found multiple reports based on a single patient cohort or overlapping cohorts. When this was encountered, we included only one of these papers, with selection based on the following hierarchy of priorities: the publication with the most comprehensive presentation of information with the most leiomyosarcomas, the largest number of patients, or the one that was the most recent. Studies in which “sarcomas” or “malignancies” were found but were not specified as “leiomyosarcoma” were excluded. The first study adequate for inclusion was published in July, 1984; the final was published in September, 2014.

**Fig. 1:**
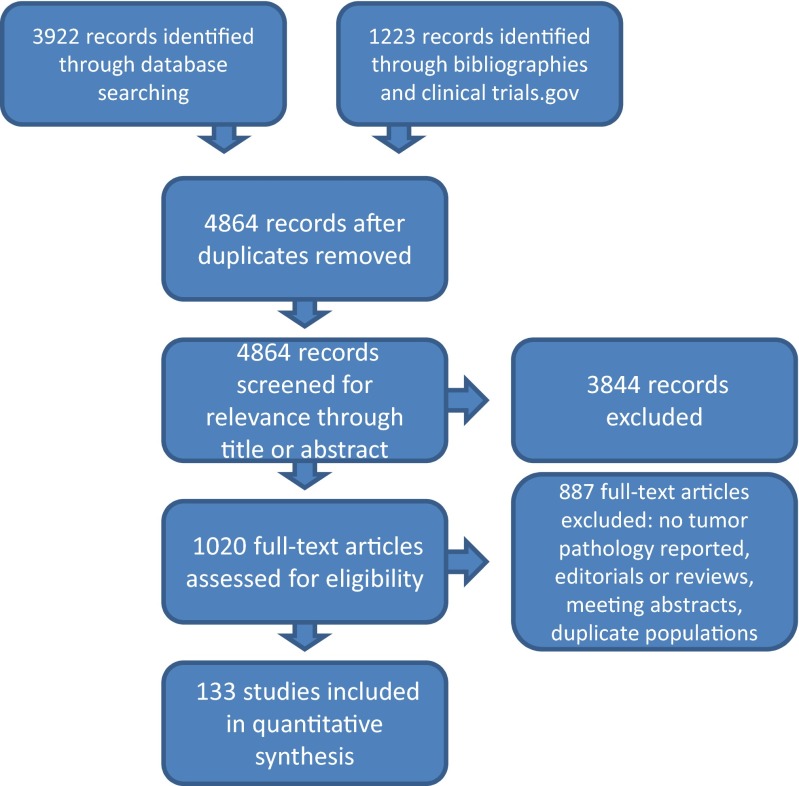
PRISMA evaluation of studies

After a thorough search of the literature, 4864 candidate studies were found. Of these, 3844 were excluded after review of the abstract. The remaining 1020 manuscripts were reviewed in their entirety. Of these, 887 were excluded after not meeting the inclusion and exclusion criteria above. One hundred thirty-three publications with 134 analyses (1 publication included both retrospective and prospective data) comprised our evidence base and were used in the final analysis as they contained postoperative histopathologic information for all reported patients (Fig. 1) (Supplemental Digital Content 1: Tables of all included studies and their characteristics).

### Statistical methods

We conducted our meta-analysis using a Bayesian binomial random effect specification (R 3.1.1; JAGS 3.3; Supplemental Digital Content 2: Bayesian statistical details and model code). We estimated separate models for prospective and retrospective studies and a model combining both study types. Inference was performed directly on posterior samples generated by Markov chain Monte Carlo. We calculated the rate of leiomyosarcoma per 1000 cases using the posterior random effect mean and constructed 95 % credible intervals (CrIs) using the posterior 2.5 and 97.5 percentiles and on the probability scale by applying the logistic retransformation to the posterior mean of the random effect mean parameter. We assessed heterogeneity on the log-odds scale by calculating the posterior mean of the random effect variance parameter *τ*^2^ and on the probability scale by applying the logistic transformation to the posterior mean of the random effect mean parameter μ_α_ ± 1.96*τ*.

For comparison with the FDA analysis, we used an unadjusted binomial mixed model with exact 95 % confidence intervals (CIs) (PROC GLIMMIX SAS 9.4).

Sensitivity analysis was conducted to determine whether the conclusions were robust in the presence of small numerical changes in events (leiomyosarcomas).

## Results

- Rate of occult leiomyosarcoma in surgery for presumed fibroids

Sixty-four published prospective analyses were included in this review: 38 as prospective cohorts [4–41] and 26 as part of a randomized clinical trial [42–67]. Thirteen studies contained more than 100 subjects, 34 included 25–99 subjects, and six had less than 25 subjects. Thirty-five studies were limited to myomectomies, 24 involved only hysterectomies, four studies included patients having either, and one did not state the type of surgery (Table 1). These analyses encompassed 5223 women, with three leiomyosarcomas being found. Only two prospective analyses found a leiomyosarcoma [34, 36].

**Table 1:** The studies: number of patients and type of surgery

	Randomized controlled studies	Prospective studies	Restrospective studies
# of patients			
>100 patients	5	8	44
25–99 patients	15	19	19
<25 patients	6	11	7
Type of surgery			
Myomectomy	12	23	25
Hysterectomy	13	11	33
Both	1	3	12
Unknown		1	

Seventy published analyses with retrospective cohorts qualified for this review, encompassing a total of 24,970 patients [33, 68–136]. Forty-four cohorts contained more than 100 women, 19 had 25–99 subjects, and seven included less than 25 women. Twenty-five reports were limited to myomectomies, 33 involved only hysterectomies, and 12 included women undergoing either (Table 1). Of these, 29 were noted to have leiomyosarcomas postsurgically. The leiomyosarcomas were found in 13 of the 70 retrospective analyses [75, 79, 84, 98, 100, 101, 106, 114, 115, 124, 125, 128, 129].

**Fig. 2:**
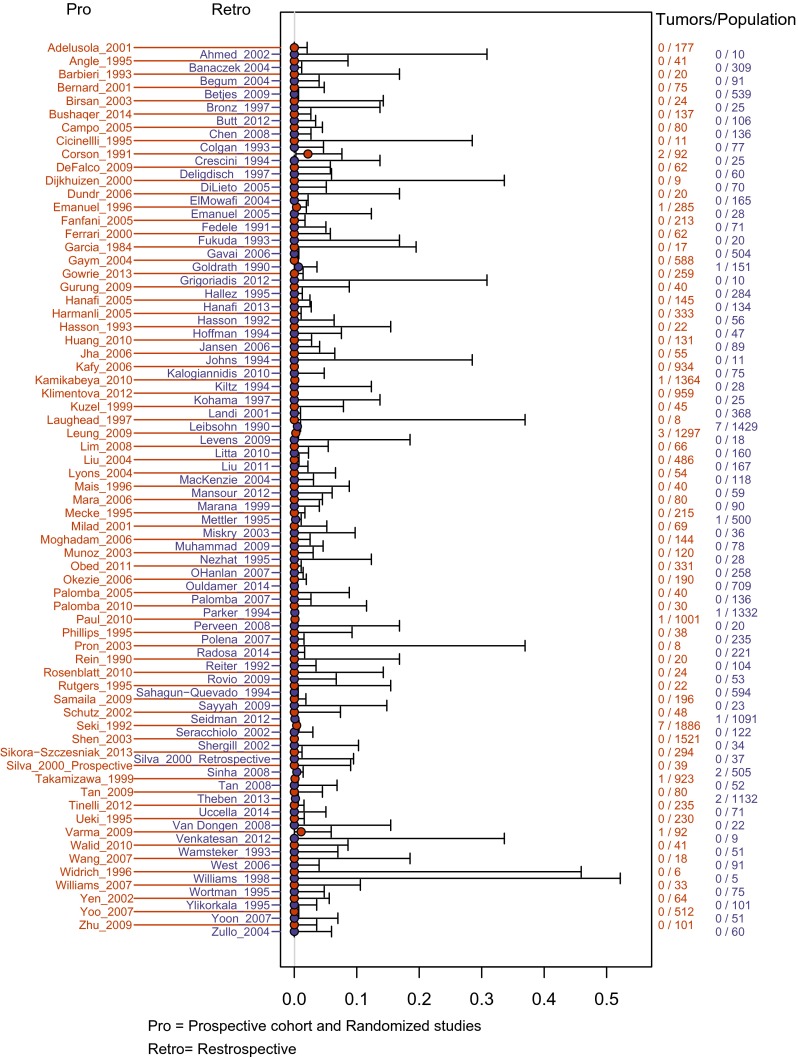
Forest plot of included studies. *Pro* prospective cohort and randomized studies, *Retro* retrospective

Taken together, these 134 analyses reported 32 leiomyosarcomas in 30,193 women undergoing surgery (Supplemental Digital Content 3: Tables of all leiomyosarcomas, sources, and their histopathology). A forest plot of these studies can be seen in Fig. 2. The meta-analysis of the 64 prospective analyses provided an estimated prevalence of leiomyosarcoma to be 0.12 per 1000 surgeries (95 % credible interval <0.01–0.75) or approximately 1 leiomyosarcoma per 8300 surgeries. When restricted to the 70 retrospective analyses, the estimated prevalence was 0.57 per 1000 surgeries (95 % CrI 0.17–1.13) or approximately 1 leiomyosarcoma per 1700 surgeries. Meta-analysis of all 134 analyses estimated prevalence to be 0.51 per 1000 surgeries (95 % CrI 0.16–0.98) or approximately 1 leiomyosarcoma for every 2000 procedures (Table 2). The posterior mean of the random effect variance parameter *τ*^2^ = 1.375, which implies that there is 95 % probability that the 134 underlying true study-specific rates of LMS ranged between 0.09 and 4.50 per 1000 surgeries.

**Table 2:** Meta-analyses of evidence base Bayesian model generalized linear mixed model

Dataset	Number of studies	Posterior mean; rate per 1000 surgeries	95 % credible interval	Rate per 1000 surgeries	95 % confidence interval
All studies	133	0.51	0.16, 0.98	0.79	0.5, 1.26
Prospective studies	64	0.12	0, 0.75	0.48	0.14, 1.72
Retrospective studies	70	0.57	0.17, 1.13	0.87	0.52, 1.46
All studies, *N* ≥ 100	57	0.55	0.17, 1.06	0.81	0.49, 1.33
Prospective studies, *N* ≥ 100	13	0.06	0, 0.62	0.45	0.06, 3.15
Retrospective studies, *N* ≥ 100	44	0.59	0.18, 1.15	0.85	0.5, 1.45
FDA dataset	9	1.86	0.7, 3.32	2.02	1.06, 3.84

b. Sensitivity analysis

Sensitivity of our analysis was tested in a variety of ways. First, seven leiomyosarcomas from three retrospective analyses uncovered in our search failed to meet current diagnostic criteria. We correctly classified these seven tumors as non-malignant and reran our analysis; the resulting prevalence estimate from our complete evidence base was essentially unchanged from the previous estimate (Table 3).

**Table 3:** Sensitivity analyses

Dataset	Posterior mean rate per 1000	95 % credible interval
All studies	0.51	0.16, 0.98
All studies; add 1 LMS case to largest study	0.59	0.21, 1.08
All studies; add 1 LMS case to smallest study	0.53	0.16, 1.02
All studies; reclassification based on histopathology	0.53	0.16, 0.99
Prospective studies	0.12	0, 0.75
Prospective studies; add 1 LMS case to largest study	0.36	0, 1.27
Prospective studies; add 1 LMS case to smallest study	0.13	0, 0.89
All studies; crude test rate	1.03	0.69, 1.43
All studies; actual crude rate	1.06	0.75, 1.50

*LMS* leiomyosarcoma

See text for explanation of adding LMS cases and reclassification.

Crude test rate involved adding 32 LMS to 31 largest studies

Actual crude rate is (number of LMS/number of total surgeries) for all studies

Secondly, we tested the robustness of the estimates by adding one leiomyosarcoma to either the largest or smallest study reporting no such malignancies. This maneuver changed the estimated rate per 1000 surgeries by 0.02–0.08 for the meta-analysis of all studies and by 0.01–0.24 per 1000 cases for the meta-analysis of prospective datasets only (Table 3).

Finally, we investigated the responsiveness of our Bayesian methodology to heterogeneity of observed rates among studies by reallocating the 32 observed leiomyomas to studies in proportion to their sample size (two each to the six largest studies and one each to the next 20 largest). This maneuver minimizes heterogeneity in observed rates and therefore should yield an estimate that approaches the crude calculated rate (number of leiomyosarcomas/number of surgeries). This was in fact the case (Table 3).

## Discussion

This meta-analysis of the existing literature reveals an estimated prevalence of leiomyosarcomas in surgeries for presumed fibroids that is substantially less than that previously estimated. For this reason, it is important to take a close look at how the estimates were derived and what they mean clinically.

Rigorously conducted systematic review and meta-analysis is widely recognized as among the highest standards of evidence for informed medical decision-making [137]. When assessing the rate of rare events, formal meta-analysis may offer the only reliable and accessible approach. It is often asked why crude rates calculated by summing the total number of events (in this case leiomyosarcomas) across studies and dividing by the total number of observations (surgeries) is not adequate for estimating the prevalence. The answer lies in the fact that the aggregate of populations from multiple studies is not the same as a single large population undergoing sampling. The heterogeneity among studies for inclusion and exclusion, confounders, and even definitions of risk factors and outcomes leads to tremendous bias in calculating a crude prevalence [138, 139]. In statistical terms, crude calculations are only appropriate if [1] each study was an independent and identically distributed measure of the overall population, and [2] the variance of each study’s estimate is known [140]. These conditions are rarely if ever met.

Heterogeneity among studies in a meta-analysis also dictates the type of analytic approach. When included studies investigate the same population with the same research questions and structure, a fixed effect model can be used. As the vast majority of studies in this analysis were not designed to estimate the prevalence of leiomyosarcomas in surgery for presumed fibroids, some degree of statistical heterogeneity is likely. Thus, a random effect meta-analysis, which assumes that design differences lead each study to produce rates that are different but related to the rate of the population of interest, was the approach used here [141]. The estimated random effect variance parameter *τ*^2^ = 1.375 suggests substantial heterogeneity between studies. However, a high degree of statistical variability between studies is to be expected in rare events random effect meta-analysis given the large number of studies with zero events (thus having arbitrarily negative log-odds).

Finally, there are a number of random effect models from which to choose. Our choice was to use a Bayesian binomial model, which has a number of advantages over classical meta-analysis techniques that are particularly important given the complexities of estimating rare event rates [141, 142] (for details, see supplemental digital content 2). Bayesian random effect meta-analysis has been used extensively under such conditions for clinical decision-making and policy analysis [143].

The best available estimate for the rate of occult leiomyosarcoma lies in the data collected prospectively: that gathered from randomized trials and prospective cohort studies. In these investigations, the data collection is begun at a predefined time point, consecutive cases are included avoiding selection bias and patient exclusion, and the data are uniformly collected for all surgeries throughout the duration of the study. In this review, the estimated prevalence of leiomyosarcoma using only data derived from prospective studies was 0.12 per 1000 surgeries, with a 97.5 % probability of being less than 0.75 per 1000 surgeries. Our sensitivity analysis suggests that this estimate is modestly sensitive to adding an incremental case of leiomyosarcoma to the largest study reporting zero events, as would be expected given the small number of prospective studies finding leiomyosarcomas.

Expanding the evidence to include retrospective studies yields an estimated rate of 0.51 per 1000 surgeries, with 97.5 % probability of being less than 0.98 per 1000. Retrospective data collection and analysis has a number of inherent biases, and these can affect the calculated prevalence in either direction. Data that cannot be found when doing chart reviews may not be representative of the entire study population but rather may represent an enriched sample. Prevalence would be underestimated if, for example, records of leiomyosarcomas were more frequently undiscovered because of being moved to hospital risk management files! Conversely, retrospective studies are often initiated after the discovery of an index case at an institution. If the ensuing study population then includes the index case, the resulting bias will uniformly overestimate rate of prevalence. In the case of leiomyosarcomas in fibroid surgery, this definitely occurred in at least two published studies [79, 100]. It is reasonable to suspect that other retrospective studies were also initiated in response to an index case but did not report this reason.

Our prevalence estimates differ substantially from that calculated in the FDA meta-analysis, which was 2.02 per 1000 surgeries. Our group has been asked why these differences are so profound. They can be attributed to both the base of evidence and the statistical methodology. To sort out the relative contribution of each, we applied our Bayesian methods to the FDA dataset and estimated a rate of 1.86 per 1000 surgeries (95 % CrI 0.70–3.32), which is about 8 % lower than the FDA’s rate (Table 2). Thus, while differences in methodology accounted for some of the difference in estimated rates, differences in the evidence base accounted for a much larger share.

The evidence base used in this study differed from the studies utilized by the FDA in a number of significant ways. First, our search and screen protocol identified all papers where surgery was being performed for presumed fibroids and where histopathology results were explicitly provided for every subject in the study. This strategy yielded 134 analyses in 133 published studies.

In contrast, to obtain their evidence, the FDA performed a targeted search using the search terms “uterine cancer” AND “hysterectomy or myomectomy” AND “incidental cancer or uterine prolapse, pelvic pain, uterine bleeding, and uterine fibroids.” Using uterine cancer as a required search term necessitates the presence of uterine cancer in the manuscripts available for analysis, while those studies without uterine cancers would be overlooked. Indeed, this was the case: 8/9 studies found in their search contained at least one leiomyosarcoma. Of the 133 published studies included in our review, 118 had no leiomyosarcomas and thus would not have appeared in the FDA’s targeted search.

A second difference lies in the fact that only studies with more than 100 subjects were included in the evidence base compiled by the FDA; their reasoning was that this would reduce bias from smaller studies. Recognizing the arbitrary nature of any predefined size threshold, our preferred approach was to include eligible studies of all sizes, while allowing for the Bayesian model to weigh each study according to its size and degree of statistical heterogeneity.

Nevertheless, the number of studies included in our evidence base with 100 or more observations was 57, a number far greater than that of the FDA. Restricting our meta-analysis approach to just these 57 prospective and retrospective studies resulted in a prevalence estimate essentially unchanged from our analysis of all 134 studies and approximately one-fourth that of the FDA’s estimates: 0.55 per 1000 (95 % CrI 0.17 to 1.06) (see Table 2). Applying the sample size restriction to our prospective-only dataset resulted in inclusion of 13 studies and an estimated rate of 0.06 per 1000 (95 % CrI <0.01 to 0.62). Thus, even utilizing the same arbitrary study size restriction as the FDA, our more comprehensive database significantly lowers the prevalence estimate from their original report.

Third, the FDA included only studies that exclusively examined procedures performed for presumed leiomyomas; if multiple indications were listed by the author of the study, it was excluded from their evidence base and was unavailable for analysis. However, many publications containing multiple indications for surgery contained unequivocal information about those women with a primary surgical indication of fibroids and the data were easily extractable. They were included in our evidence base if the patients undergoing hysterectomy or myomectomy for fibroids were clearly identified, if histopathology was performed on all cases, and if results were explicitly provided.

Fourth, the FDA excluded all non-English articles from consideration. We felt the inclusion of non-English publications made for a more comprehensive review of the subject and thus included studies regardless of the language of publication.

Fifth, the FDA included one non-peer-reviewed abstract [144] and one letter to the editor [145] in their dataset. We excluded these and other similar data, restricting our analysis to peer-reviewed publications containing five or more applicable subjects. Parenthetically, the letter to the editor included in the FDA evidence base was written in English [145]. The original data were reported in their entirety in a French language publication. We excluded the letter to the editor but found the original, peer-reviewed publication and included it in our evidence base. There were three leiomyosarcomas presented in this study [101].

Finally, we note that in the FDA’s review of the nine studies referenced, eight were retrospective studies [98, 100, 101, 106, 114, 124, 128, 144] and one was a report from prospectively collected data [34]. Such a preponderance of retrospective reports raises concerns of significant ascertainment bias in the resulting prevalence rate. Our analysis contained a sufficient number of prospective studies to allow analysis restricted to only these, producing what we believe to be the most appropriate evidence base from which to calculate prevalence.

An additional bias may affect the analysis in both our study and that of the FDA. Many of the publications used were from referral centers, where patients are often sent for surgery because of an increased suspicion of additional pathology; without the ability to exclude such cases from the routine hysterectomy or myomectomy for presumed fibroids, the rate of sarcoma will be overstated. This could be compounded by the known bias of non-publication of negative results. A group looking for occult sarcomas with zero events in their study would be less likely to submit for or be accepted for publication.

Despite the comprehensiveness of this review, there are still potential shortcomings of this type of assessment. First, due to the large number of publications involving surgery for uterine fibroids from around the world, it is possible that some went undiscovered by our investigation. However, the large number of studies evaluated and the breadth of contexts considered suggest that such publications are few in number. Another related concern would be that if only a few leiomyosarcomas were overlooked, the calculated prevalence might change substantially. This is unlikely, however, due to the relatively small changes in prevalence seen with our sensitivity analysis.

It is also possible that leiomyosarcomas were missed in the surgeries performed due to incomplete removal of all fibroids or inadequate histopathologic examination. While we do not have evidence to estimate this rate, we believe this to be at most a relatively rare phenomenon. Nevertheless, our sensitivity analyses suggested robustness of our results, as there were relatively small changes in estimated rates from the addition of an incremental case of leiomyosarcoma to one large or small trial previously reporting no cases. There was also a relatively small change when correctly categorizing seven benign tumors that were originally diagnosed as leiomyosarcomas.

Concern might also be expressed that the vast majority of studies included in this analysis, including all prospective studies and randomized controlled trials, were not designed to address the issue of leiomyosarcoma prevalence in such surgeries. Thus, inclusion criteria may have inadvertently eliminated many subjects who would be at higher risk for such malignancies. While this is undoubtedly the case with some trials, the large number of studies and the widely varying reasons for study performance speak against a systematic bias. Age ranges were similar for all datasets and very few restricted patient inclusion a priori to premenopausal women (Table 4). Moreover, the wide-ranging study hypotheses suggest that the information obtained is applicable to real-world clinical situations where surgery is performed for uterine fibroids.

**Table 4:** Age distributions by study type and histopathology

Dataset	Study number	Premenopausal only	Study mean ages	Age range
Randomized trials	26	10	35.8–53.4	20–70
Prospective	38	4	28.9–67.4	20–83
Retrospective	70	0	32.6–59.6	19–91
Studies with leiomyosarcomas	14	0	32.6–48.0	21–81
Leiomyosarcoma patients	–	–		30–63 17 ≤ 50 6 > 509 unknown

We note that during data extraction, studies were excluded from our analysis when they stated that all specimens were sent for histopathologic analysis, but the results were not included in the publications. In these cases, we expect that the tumors were benign, as surely an event such as an occult leiomyosarcoma would warrant reporting. Excluding such studies potentially underestimated benign cases in our study, but we believe that our conservative approach and rigorous inclusion criteria increase the credibility of our prevalence rate.

A final issue worth noting is that of the criteria for the diagnosis of leiomyosarcoma. The criteria used today for leiomyosarcoma are those adopted by the World Health Organization in 2003 [3]. These criteria indicate that a malignant neoplasm composed of cells demonstrating uterine smooth muscle differentiation with coagulative tumor cell necrosis (not hyaline necrosis) is a leiomyosarcoma. If no such necrosis exists, then the diagnosis is made only if the mitotic index is ≥10 mitoses per 10 high-power fields and there is diffuse, moderate to severe cytologic atypia. The microscopic criteria to meet each of the three requirements are quite specific.

Many of the leiomyosarcomas found in our search provided histologic detail in the manuscript. Interestingly, 7 of the 32 “leiomyosarcomas” found in our search would, based on current WHO criteria, not be so classified today (Table 5). Further validation of their non-malignant nature is found in the fact that none of the seven had recurrence following surgery. Despite convincing evidence that these seven tumors were not in fact leiomyosarcomas, we have maintained the original diagnosis in our calculations. Our sensitivity analysis suggests these mislabeled tumors had little impact on the estimated prevalence. Nevertheless, this factor highlights a potential bias in utilizing data from older or less highly scrutinized studies: the potential for overestimating prevalence of clinically relevant leiomyosarcomas via misinterpretation of histopathology. Our search for this review included manuscripts published after 1960, in an attempt to be as all-inclusive as possible. We found no studies that met inclusion criteria between 1960 and 1983. The FDA’s inclusion dates for studies were between 1980 and 2014, making comparison of these two analyses justifiable. However, both reviews are likely to be overstating the number of actual leiomyosarcomas uncovered.

**Table 5:** Tumors inconsistent with World Health Organization 2003 leiomyosarcoma criteria

Author/date type	Leiomyoma sub-type	Age (years)	Pathology	Recurrence
∞Leibsohn/1990 retro	Atypical	36	6 mitoses/10 HPF, “poorly demarcated,” cellular atypia	NED 6 months
	Atypical	48	7 mitoses/10 HPF, cellular atypia	NED 16 months
∞Parker/1994 retro	Atypical	30	Irregular infiltrative borders, mild nuclear atypia, 5–8 mitoses/10 HPF	NED “years”
Seki/1992 retro	Mitotically active	33	6 mitoses/10 HPF, no cellular atypia	NED 11 months
	Mitotically active	34	5 mitoses/10 HPF, no cellular atypia	NED 57 months
	Mitotically active	43	8 mitoses/10 HPF, no cellular atypia	NED 61 months
	Mitotically active	43	9 mitoses/10 HPF, no cellular atypia	NED 72 months

*HPF* high-powered field

*Retro* retrospective

*∞* included in FDA analysis

*NED* no evidence of disease

While we have found that the prevalence of occult leiomyosarcoma is less than previously estimated, this does not negate the fact that such occult malignancies can and do occur. Furthermore, a number of other malignancies have been found at these surgical procedures. It is ideal to diagnose a tumor accurately prior to deciding the type of surgery that is appropriate. The more common types of uterine cancers may be diagnosed preoperatively, but there is no accurate way to do so for leiomyosarcomas. Many uterine leiomyosarcomas, particularly in younger women, are diagnosed after the tumor has been removed surgically. It was beyond the scope of this analysis to detail or quantify the risk of other cancers in surgery for presumed fibroids, but investigation should continue for a more thorough elucidation of the risks of all such tumors as well as the relative benefits of different surgical approaches. We believe that such data will allow more meaningful research into the decision analysis required for this complex clinical issue.

## Electronic supplementary material

Supplemental Digital Content 1 A series of tables describing all included studies. The studies are sorted by those with a retrospective design (Table 1), prospective cohort design (Table 2), and randomized clinical trials (Table 3). The tables contain the number of subjects, age, number of leiomyosarcomas, indication for surgery, and type of surgery for each study. (PPTX 83 kb)

Supplemental Digital Content 2 A detailed description of the Bayesian model used in this analysis, including the code used for running the data. (DOCX 40 kb)

Supplemental Digital Content 3 Two tables detailing information regarding leiomyosarcomas uncovered by this search. Table 1 provides details of the studies in which leiomyosarcomas were found and the number found, presence or absence of histopathology, age, data collection period, whether or not morcellation occurred, and outcome. Table 2 provides detail of the reported histopathology of each leiomyosarcoma. (PPTX 70 kb)
