# Supplementary material for: The prevalence of occult leiomyosarcoma at surgery for presumed uterine fibroids: a meta-analysis
Source: Gynecol Surg. 2015 May 19;12(3):165–77. doi: 10.1007/s10397-015-0894-4 (PMC4532723; doi:10.1007/s10397-015-0894-4)
Supplement: Supplementary file 1 — A series of tables describing all included studies. The studies are sorted by those with a retrospective design (Table 1), prospective cohort design (Table 2), and randomized clinical trials (Table 3). The tables contain the number of subjects, age, number of leiomyosarcomas, indication for surgery, and type of surgery for each study. (PPTX 83 kb) [file 10397_2015_894_MOESM1_ESM.pptx]

## Slide 1
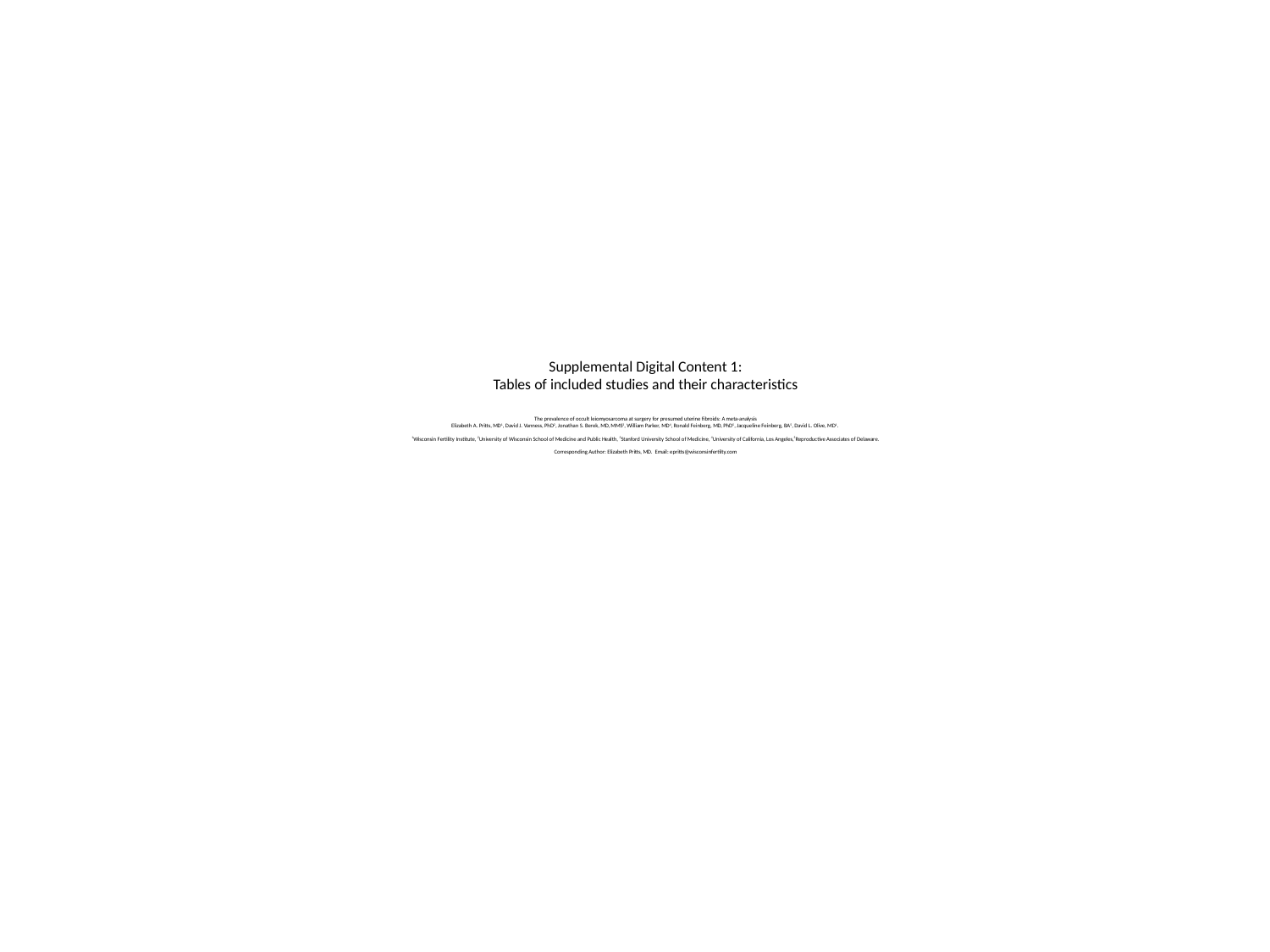

# Supplemental Digital Content 1: Tables of included studies and their characteristics The prevalence of occult leiomyosarcoma at surgery for presumed uterine fibroids: A meta-analysis Elizabeth A. Pritts, MD1, David J. Vanness, PhD2, Jonathan S. Berek, MD, MMS3, William Parker, MD4, Ronald Feinberg, MD, PhD5, Jacqueline Feinberg, BA5, David L. Olive, MD1.   1Wisconsin Fertility Institute, 2University of Wisconsin School of Medicine and Public Health, 3Stanford University School of Medicine, 4University of California, Los Angeles,5Reproductive Associates of Delaware.   Corresponding Author: Elizabeth Pritts, MD. Email: epritts@wisconsinfertilty.com

## Slide 2
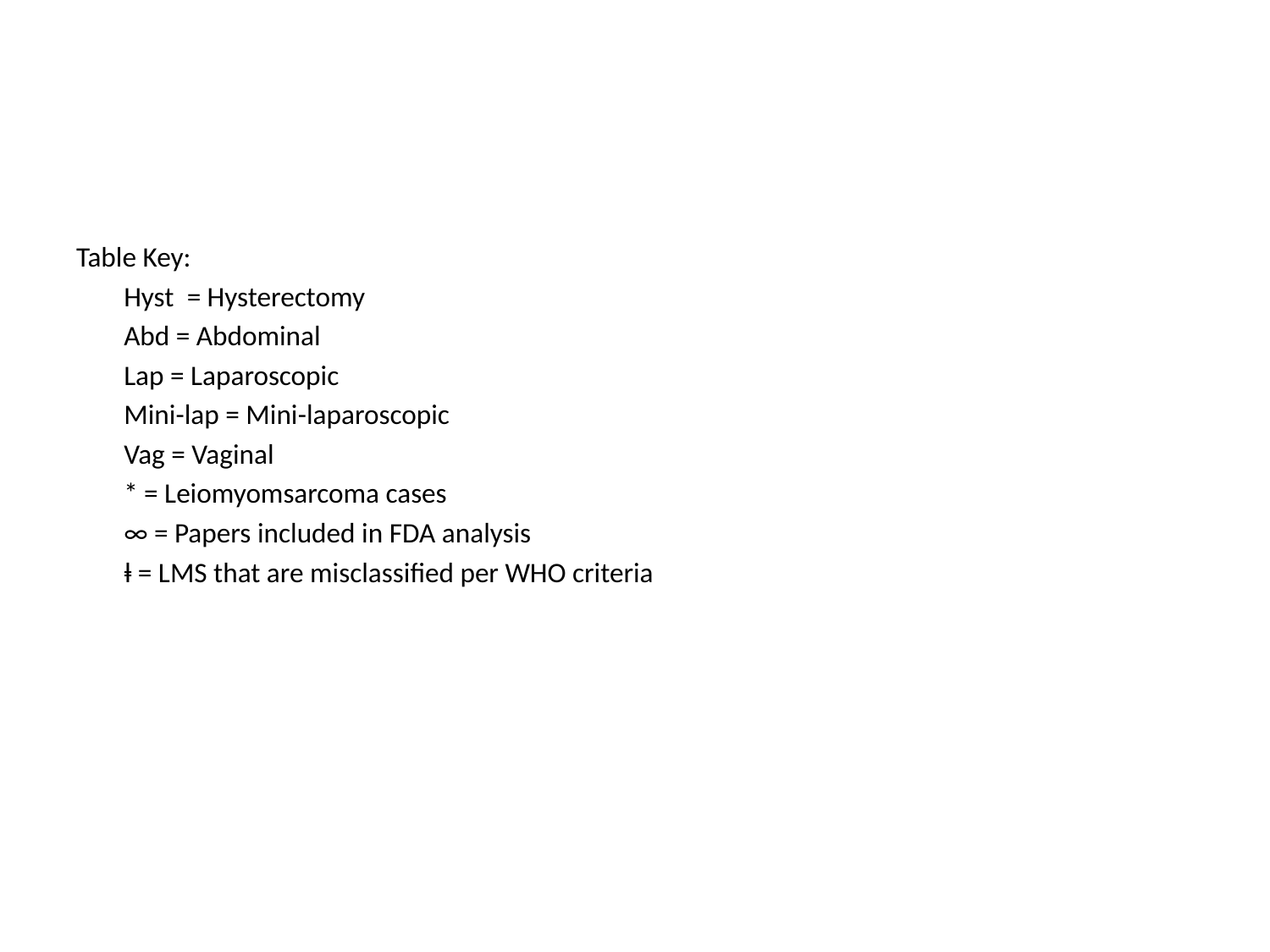

Table Key:
	Hyst = Hysterectomy
	Abd = Abdominal
	Lap = Laparoscopic
	Mini-lap = Mini-laparoscopic
	Vag = Vaginal
	* = Leiomyomsarcoma cases
	∞ = Papers included in FDA analysis
	ⱡ = LMS that are misclassified per WHO criteria

## Slide 3
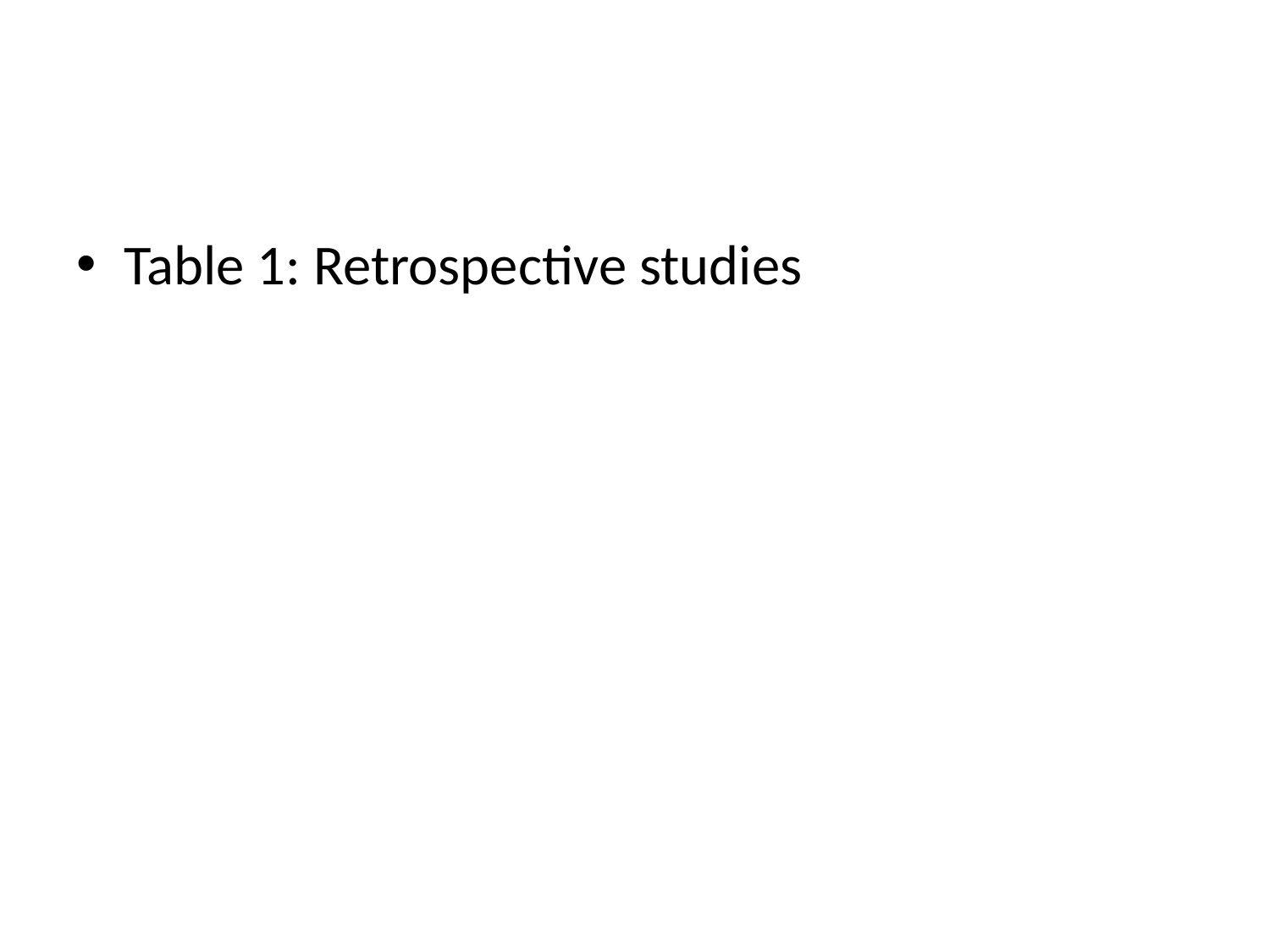

Table 1: Retrospective studies

## Slide 4
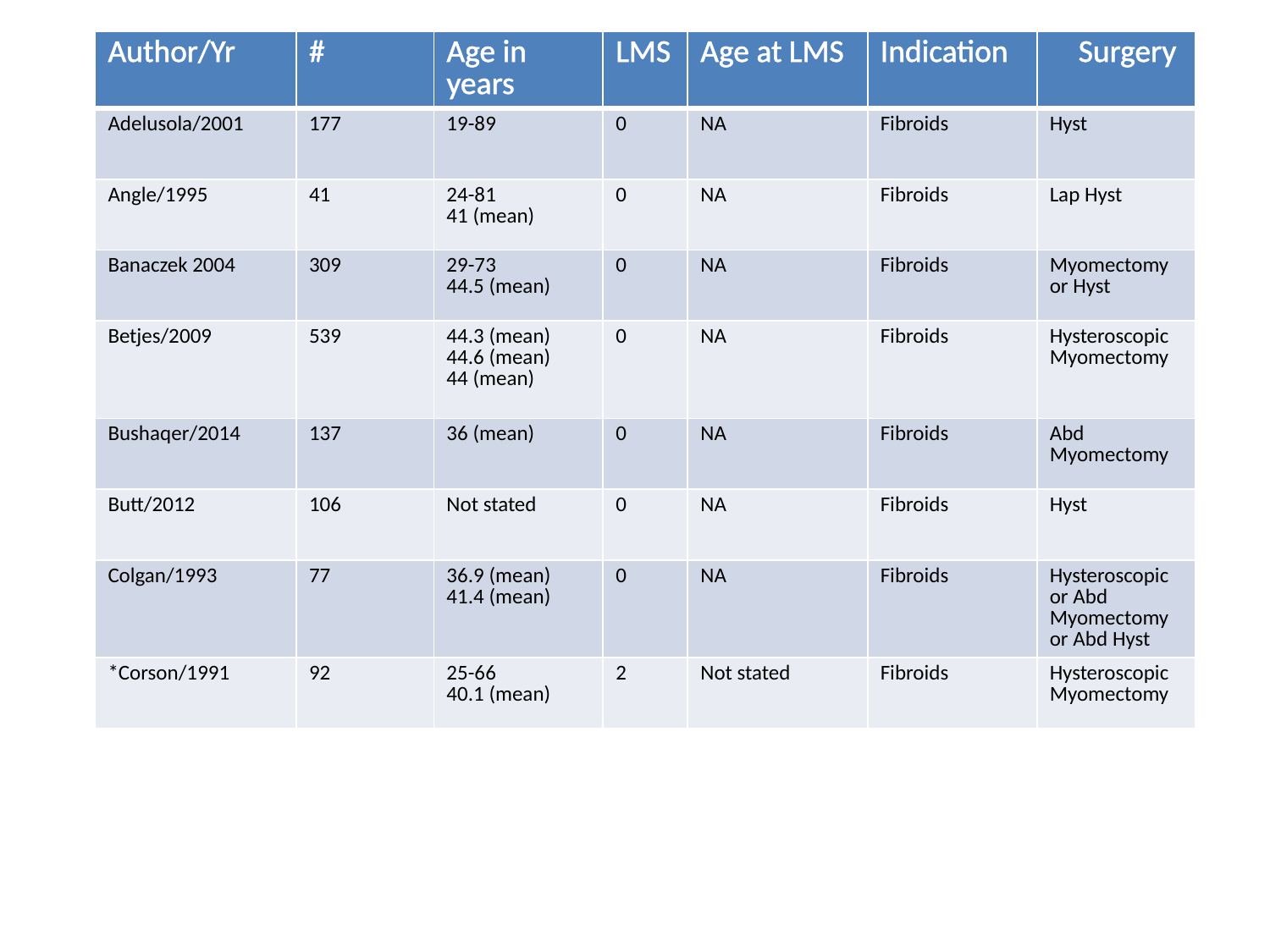

| Author/Yr | # | Age in years | LMS | Age at LMS | Indication | Surgery |
| --- | --- | --- | --- | --- | --- | --- |
| Adelusola/2001 | 177 | 19-89 | 0 | NA | Fibroids | Hyst |
| Angle/1995 | 41 | 24-81 41 (mean) | 0 | NA | Fibroids | Lap Hyst |
| Banaczek 2004 | 309 | 29-73 44.5 (mean) | 0 | NA | Fibroids | Myomectomy or Hyst |
| Betjes/2009 | 539 | 44.3 (mean) 44.6 (mean) 44 (mean) | 0 | NA | Fibroids | Hysteroscopic Myomectomy |
| Bushaqer/2014 | 137 | 36 (mean) | 0 | NA | Fibroids | Abd Myomectomy |
| Butt/2012 | 106 | Not stated | 0 | NA | Fibroids | Hyst |
| Colgan/1993 | 77 | 36.9 (mean) 41.4 (mean) | 0 | NA | Fibroids | Hysteroscopic or Abd Myomectomy or Abd Hyst |
| \*Corson/1991 | 92 | 25-66 40.1 (mean) | 2 | Not stated | Fibroids | Hysteroscopic Myomectomy |
#

## Slide 5
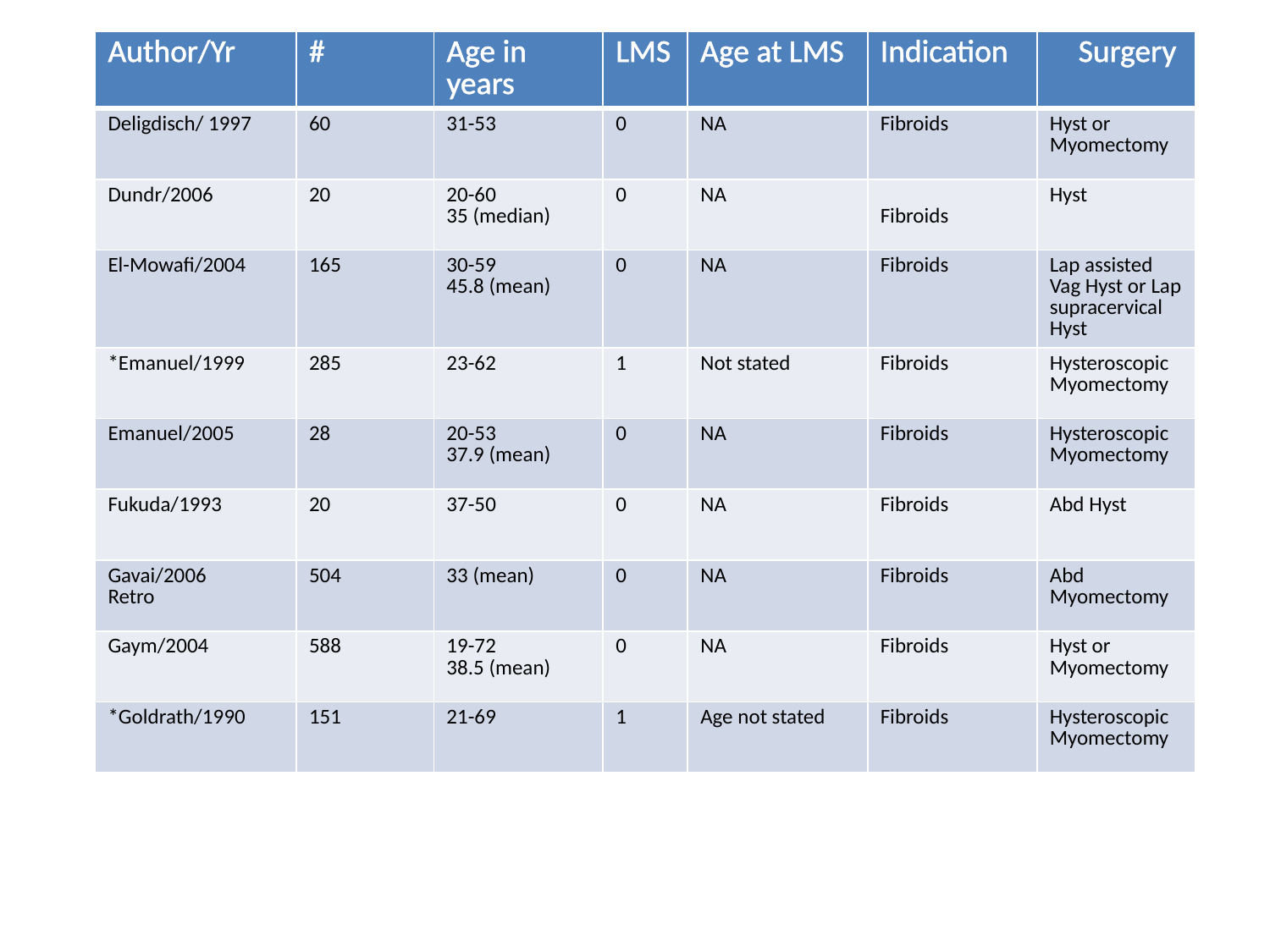

| Author/Yr | # | Age in years | LMS | Age at LMS | Indication | Surgery |
| --- | --- | --- | --- | --- | --- | --- |
| Deligdisch/ 1997 | 60 | 31-53 | 0 | NA | Fibroids | Hyst or Myomectomy |
| Dundr/2006 | 20 | 20-60 35 (median) | 0 | NA | Fibroids | Hyst |
| El-Mowafi/2004 | 165 | 30-59 45.8 (mean) | 0 | NA | Fibroids | Lap assisted Vag Hyst or Lap supracervical Hyst |
| \*Emanuel/1999 | 285 | 23-62 | 1 | Not stated | Fibroids | Hysteroscopic Myomectomy |
| Emanuel/2005 | 28 | 20-53 37.9 (mean) | 0 | NA | Fibroids | Hysteroscopic Myomectomy |
| Fukuda/1993 | 20 | 37-50 | 0 | NA | Fibroids | Abd Hyst |
| Gavai/2006 Retro | 504 | 33 (mean) | 0 | NA | Fibroids | Abd Myomectomy |
| Gaym/2004 | 588 | 19-72 38.5 (mean) | 0 | NA | Fibroids | Hyst or Myomectomy |
| \*Goldrath/1990 | 151 | 21-69 | 1 | Age not stated | Fibroids | Hysteroscopic Myomectomy |
#

## Slide 6
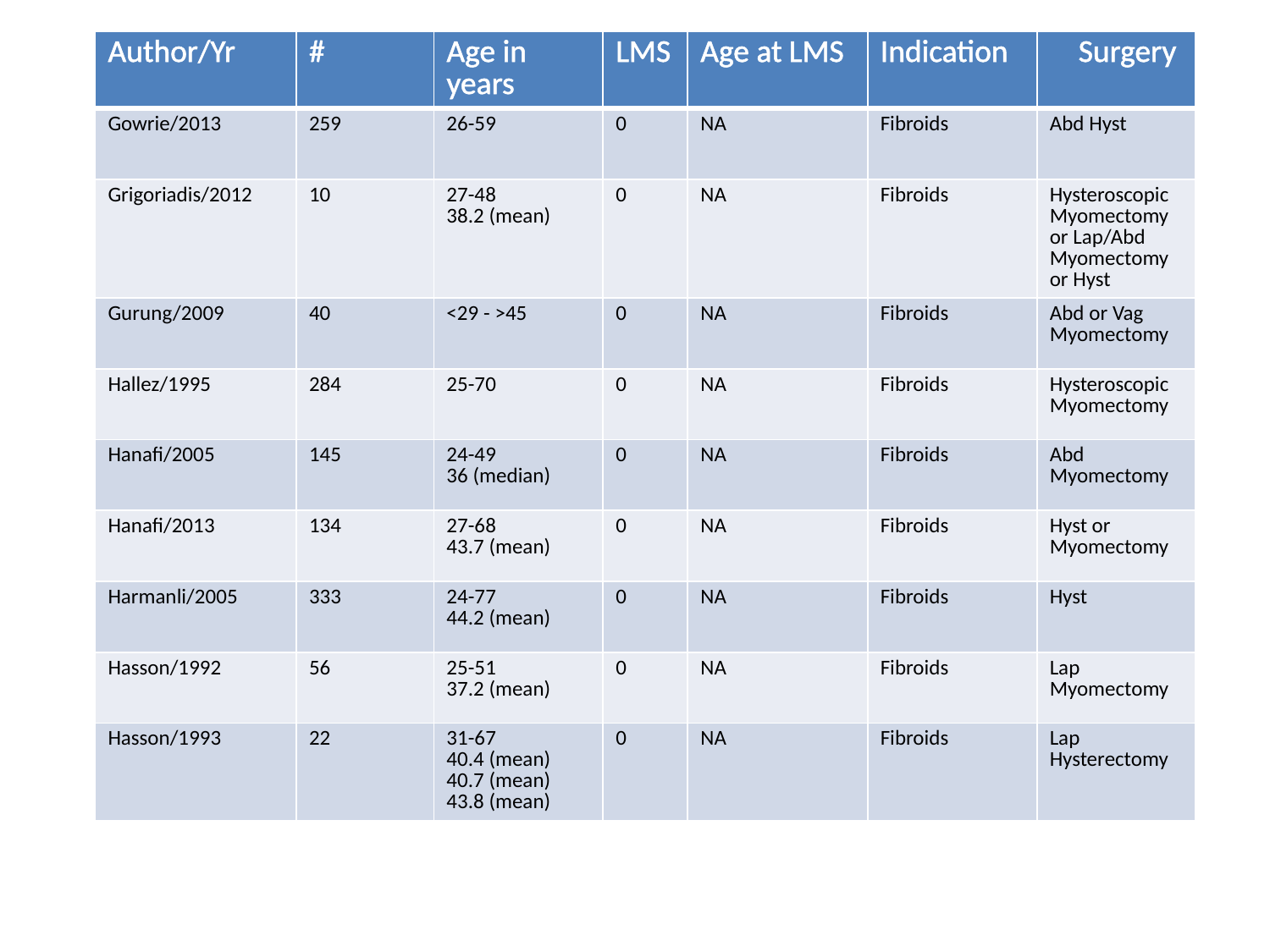

| Author/Yr | # | Age in years | LMS | Age at LMS | Indication | Surgery |
| --- | --- | --- | --- | --- | --- | --- |
| Gowrie/2013 | 259 | 26-59 | 0 | NA | Fibroids | Abd Hyst |
| Grigoriadis/2012 | 10 | 27-48 38.2 (mean) | 0 | NA | Fibroids | Hysteroscopic Myomectomy or Lap/Abd Myomectomy or Hyst |
| Gurung/2009 | 40 | <29 - >45 | 0 | NA | Fibroids | Abd or Vag Myomectomy |
| Hallez/1995 | 284 | 25-70 | 0 | NA | Fibroids | Hysteroscopic Myomectomy |
| Hanafi/2005 | 145 | 24-49 36 (median) | 0 | NA | Fibroids | Abd Myomectomy |
| Hanafi/2013 | 134 | 27-68 43.7 (mean) | 0 | NA | Fibroids | Hyst or Myomectomy |
| Harmanli/2005 | 333 | 24-77 44.2 (mean) | 0 | NA | Fibroids | Hyst |
| Hasson/1992 | 56 | 25-51 37.2 (mean) | 0 | NA | Fibroids | Lap Myomectomy |
| Hasson/1993 | 22 | 31-67 40.4 (mean) 40.7 (mean) 43.8 (mean) | 0 | NA | Fibroids | Lap Hysterectomy |
#

## Slide 7
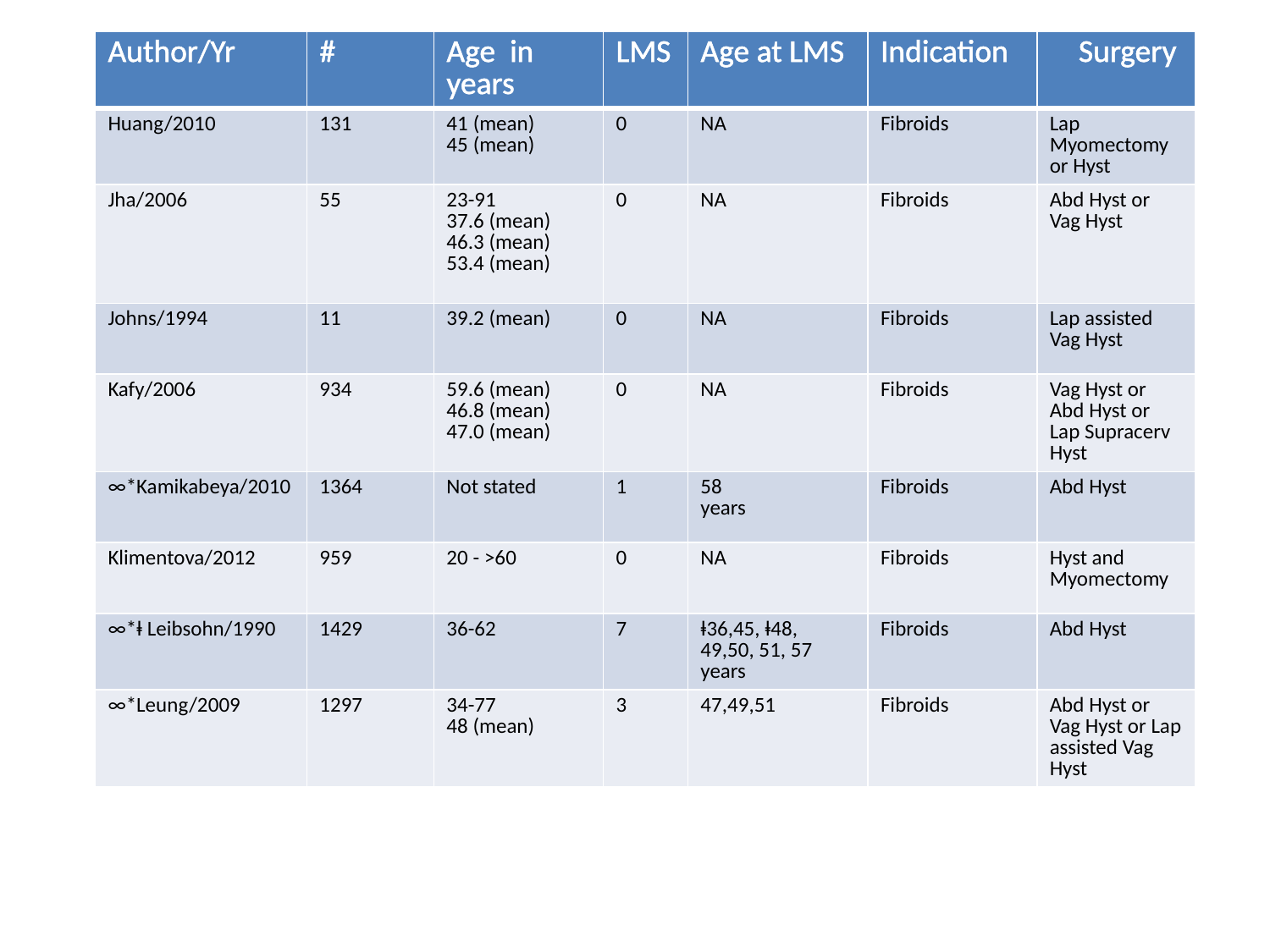

| Author/Yr | # | Age in years | LMS | Age at LMS | Indication | Surgery |
| --- | --- | --- | --- | --- | --- | --- |
| Huang/2010 | 131 | 41 (mean) 45 (mean) | 0 | NA | Fibroids | Lap Myomectomy or Hyst |
| Jha/2006 | 55 | 23-91 37.6 (mean) 46.3 (mean) 53.4 (mean) | 0 | NA | Fibroids | Abd Hyst or Vag Hyst |
| Johns/1994 | 11 | 39.2 (mean) | 0 | NA | Fibroids | Lap assisted Vag Hyst |
| Kafy/2006 | 934 | 59.6 (mean) 46.8 (mean) 47.0 (mean) | 0 | NA | Fibroids | Vag Hyst or Abd Hyst or Lap Supracerv Hyst |
| ∞\*Kamikabeya/2010 | 1364 | Not stated | 1 | 58 years | Fibroids | Abd Hyst |
| Klimentova/2012 | 959 | 20 - >60 | 0 | NA | Fibroids | Hyst and Myomectomy |
| ∞\*ⱡ Leibsohn/1990 | 1429 | 36-62 | 7 | ⱡ36,45, ⱡ48, 49,50, 51, 57 years | Fibroids | Abd Hyst |
| ∞\*Leung/2009 | 1297 | 34-77 48 (mean) | 3 | 47,49,51 | Fibroids | Abd Hyst or Vag Hyst or Lap assisted Vag Hyst |
#

## Slide 8
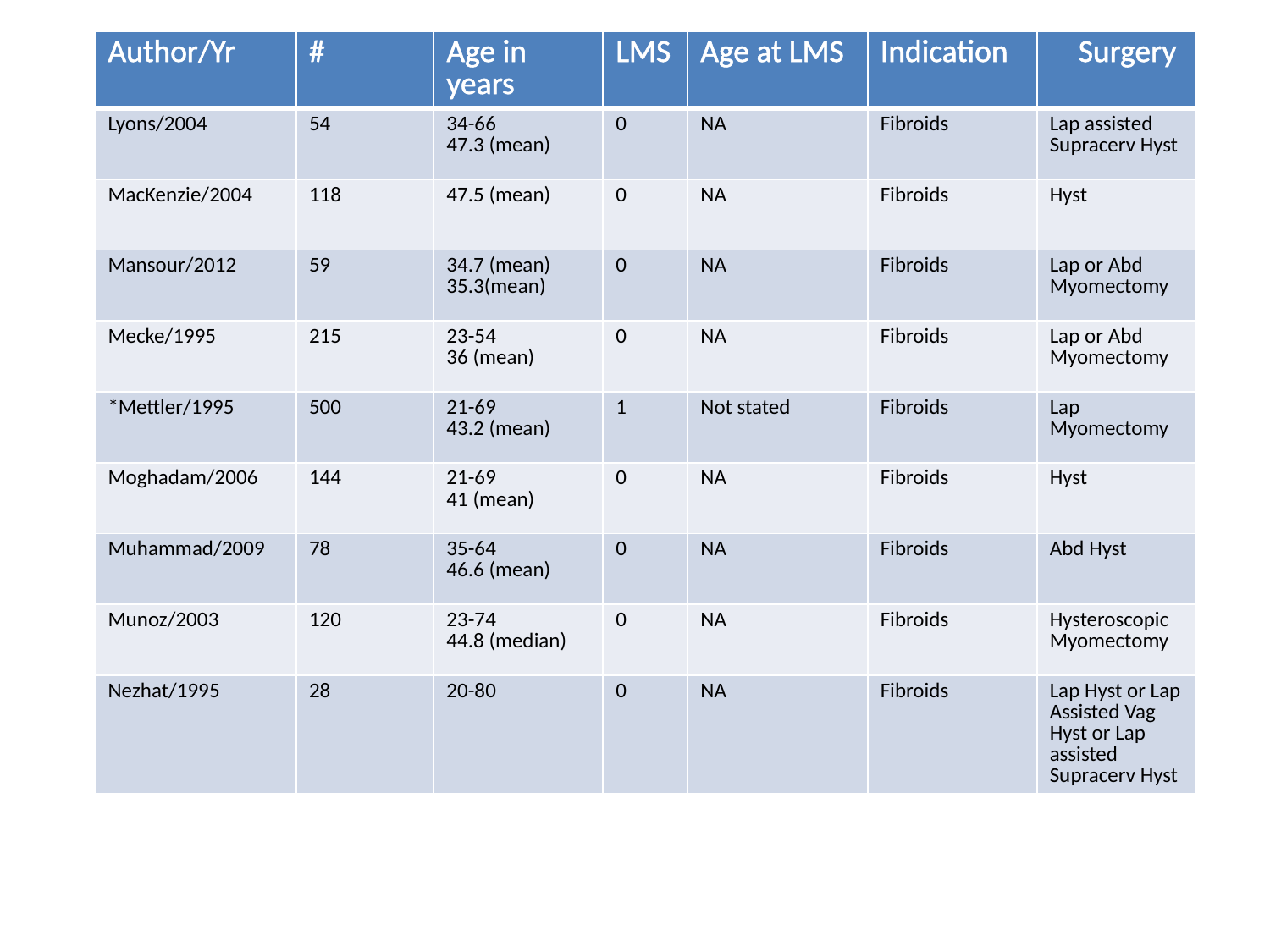

| Author/Yr | # | Age in years | LMS | Age at LMS | Indication | Surgery |
| --- | --- | --- | --- | --- | --- | --- |
| Lyons/2004 | 54 | 34-66 47.3 (mean) | 0 | NA | Fibroids | Lap assisted Supracerv Hyst |
| MacKenzie/2004 | 118 | 47.5 (mean) | 0 | NA | Fibroids | Hyst |
| Mansour/2012 | 59 | 34.7 (mean) 35.3(mean) | 0 | NA | Fibroids | Lap or Abd Myomectomy |
| Mecke/1995 | 215 | 23-54 36 (mean) | 0 | NA | Fibroids | Lap or Abd Myomectomy |
| \*Mettler/1995 | 500 | 21-69 43.2 (mean) | 1 | Not stated | Fibroids | Lap Myomectomy |
| Moghadam/2006 | 144 | 21-69 41 (mean) | 0 | NA | Fibroids | Hyst |
| Muhammad/2009 | 78 | 35-64 46.6 (mean) | 0 | NA | Fibroids | Abd Hyst |
| Munoz/2003 | 120 | 23-74 44.8 (median) | 0 | NA | Fibroids | Hysteroscopic Myomectomy |
| Nezhat/1995 | 28 | 20-80 | 0 | NA | Fibroids | Lap Hyst or Lap Assisted Vag Hyst or Lap assisted Supracerv Hyst |
#

## Slide 9
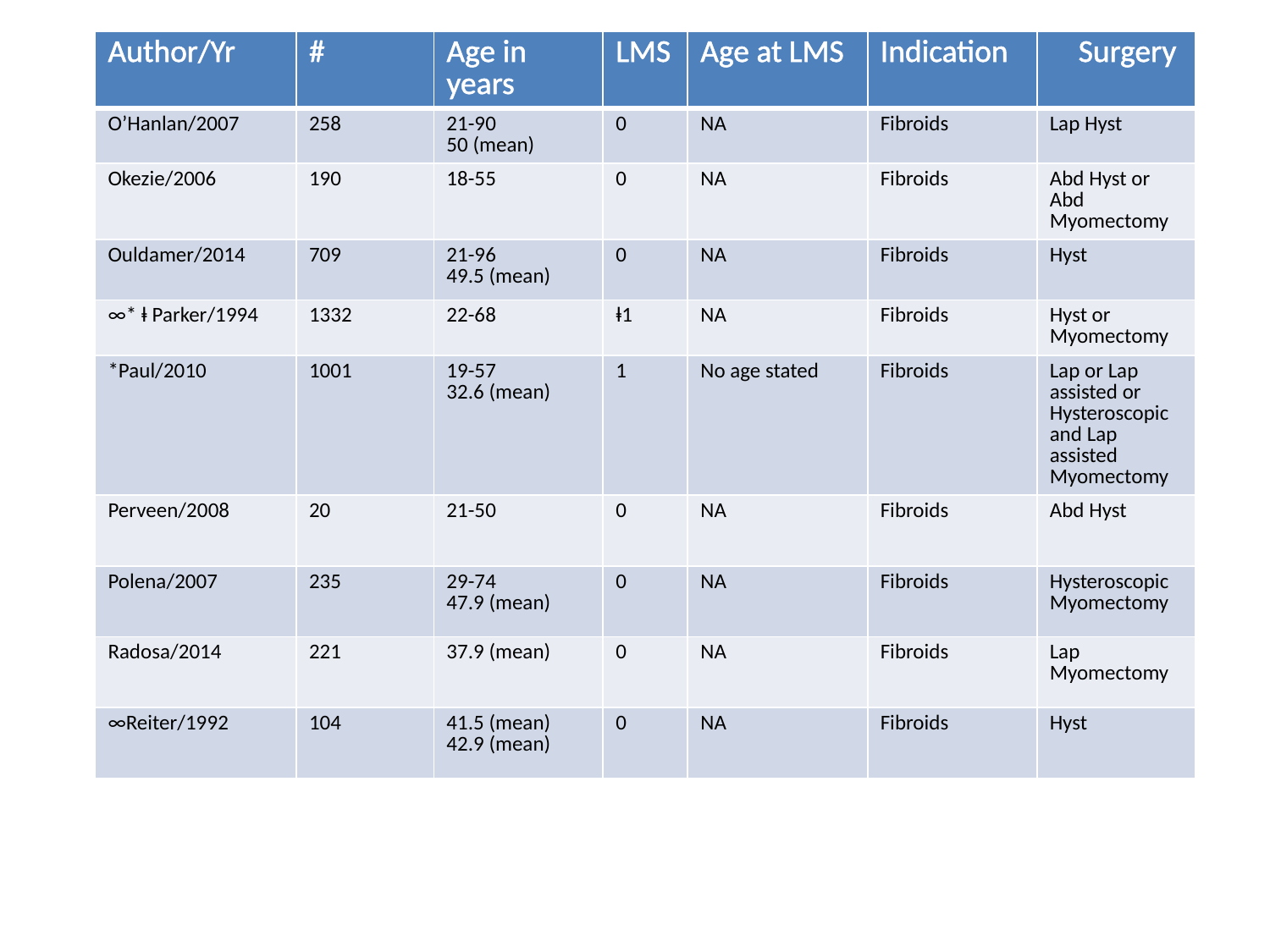

| Author/Yr | # | Age in years | LMS | Age at LMS | Indication | Surgery |
| --- | --- | --- | --- | --- | --- | --- |
| O’Hanlan/2007 | 258 | 21-90 50 (mean) | 0 | NA | Fibroids | Lap Hyst |
| Okezie/2006 | 190 | 18-55 | 0 | NA | Fibroids | Abd Hyst or Abd Myomectomy |
| Ouldamer/2014 | 709 | 21-96 49.5 (mean) | 0 | NA | Fibroids | Hyst |
| ∞\* ⱡ Parker/1994 | 1332 | 22-68 | ⱡ1 | NA | Fibroids | Hyst or Myomectomy |
| \*Paul/2010 | 1001 | 19-57 32.6 (mean) | 1 | No age stated | Fibroids | Lap or Lap assisted or Hysteroscopic and Lap assisted Myomectomy |
| Perveen/2008 | 20 | 21-50 | 0 | NA | Fibroids | Abd Hyst |
| Polena/2007 | 235 | 29-74 47.9 (mean) | 0 | NA | Fibroids | Hysteroscopic Myomectomy |
| Radosa/2014 | 221 | 37.9 (mean) | 0 | NA | Fibroids | Lap Myomectomy |
| ∞Reiter/1992 | 104 | 41.5 (mean) 42.9 (mean) | 0 | NA | Fibroids | Hyst |
#

## Slide 10
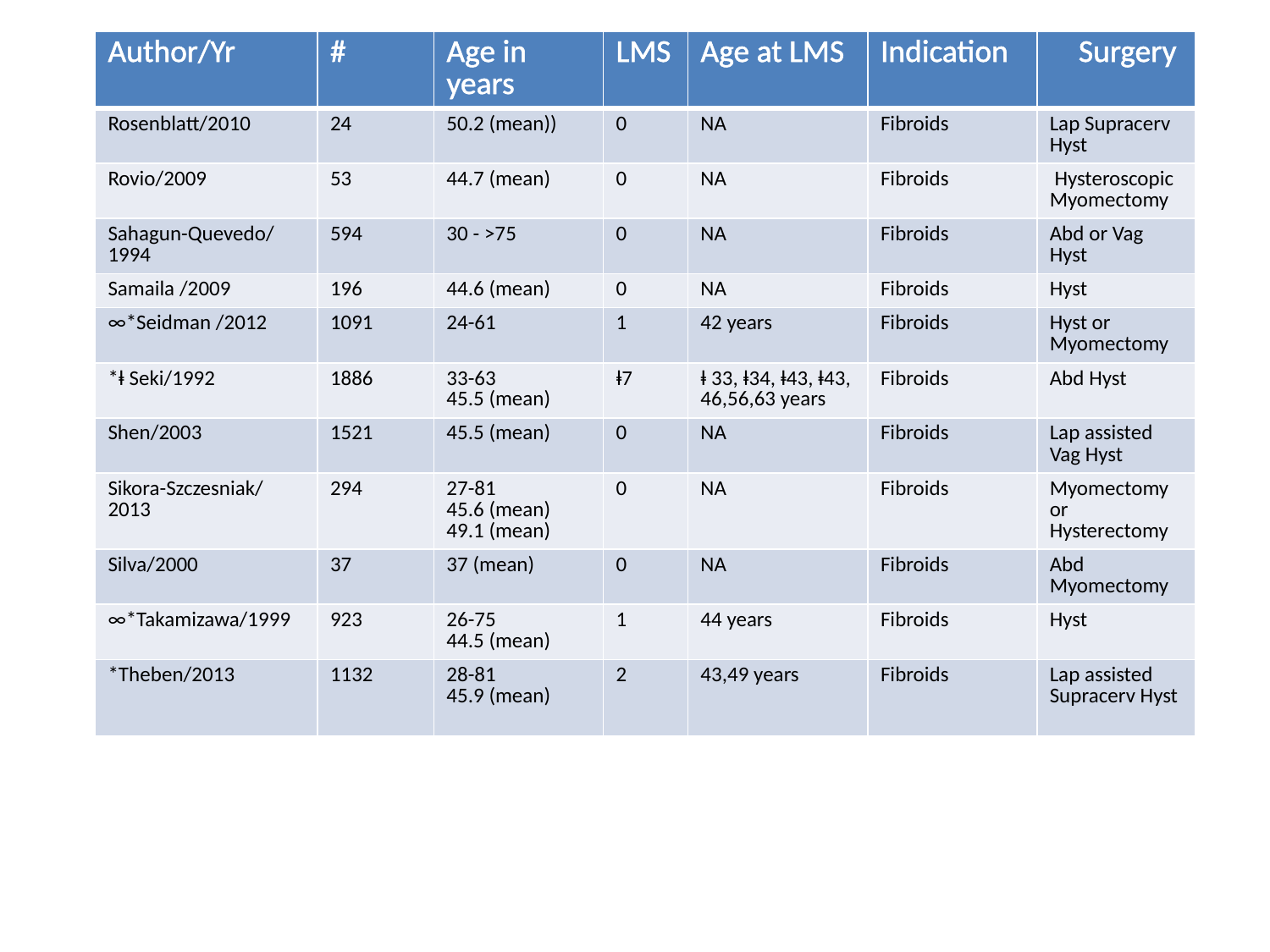

| Author/Yr | # | Age in years | LMS | Age at LMS | Indication | Surgery |
| --- | --- | --- | --- | --- | --- | --- |
| Rosenblatt/2010 | 24 | 50.2 (mean)) | 0 | NA | Fibroids | Lap Supracerv Hyst |
| Rovio/2009 | 53 | 44.7 (mean) | 0 | NA | Fibroids | Hysteroscopic Myomectomy |
| Sahagun-Quevedo/ 1994 | 594 | 30 - >75 | 0 | NA | Fibroids | Abd or Vag Hyst |
| Samaila /2009 | 196 | 44.6 (mean) | 0 | NA | Fibroids | Hyst |
| ∞\*Seidman /2012 | 1091 | 24-61 | 1 | 42 years | Fibroids | Hyst or Myomectomy |
| \*ⱡ Seki/1992 | 1886 | 33-63 45.5 (mean) | ⱡ7 | ⱡ 33, ⱡ34, ⱡ43, ⱡ43, 46,56,63 years | Fibroids | Abd Hyst |
| Shen/2003 | 1521 | 45.5 (mean) | 0 | NA | Fibroids | Lap assisted Vag Hyst |
| Sikora-Szczesniak/ 2013 | 294 | 27-81 45.6 (mean) 49.1 (mean) | 0 | NA | Fibroids | Myomectomy or Hysterectomy |
| Silva/2000 | 37 | 37 (mean) | 0 | NA | Fibroids | Abd Myomectomy |
| ∞\*Takamizawa/1999 | 923 | 26-75 44.5 (mean) | 1 | 44 years | Fibroids | Hyst |
| \*Theben/2013 | 1132 | 28-81 45.9 (mean) | 2 | 43,49 years | Fibroids | Lap assisted Supracerv Hyst |
#

## Slide 11
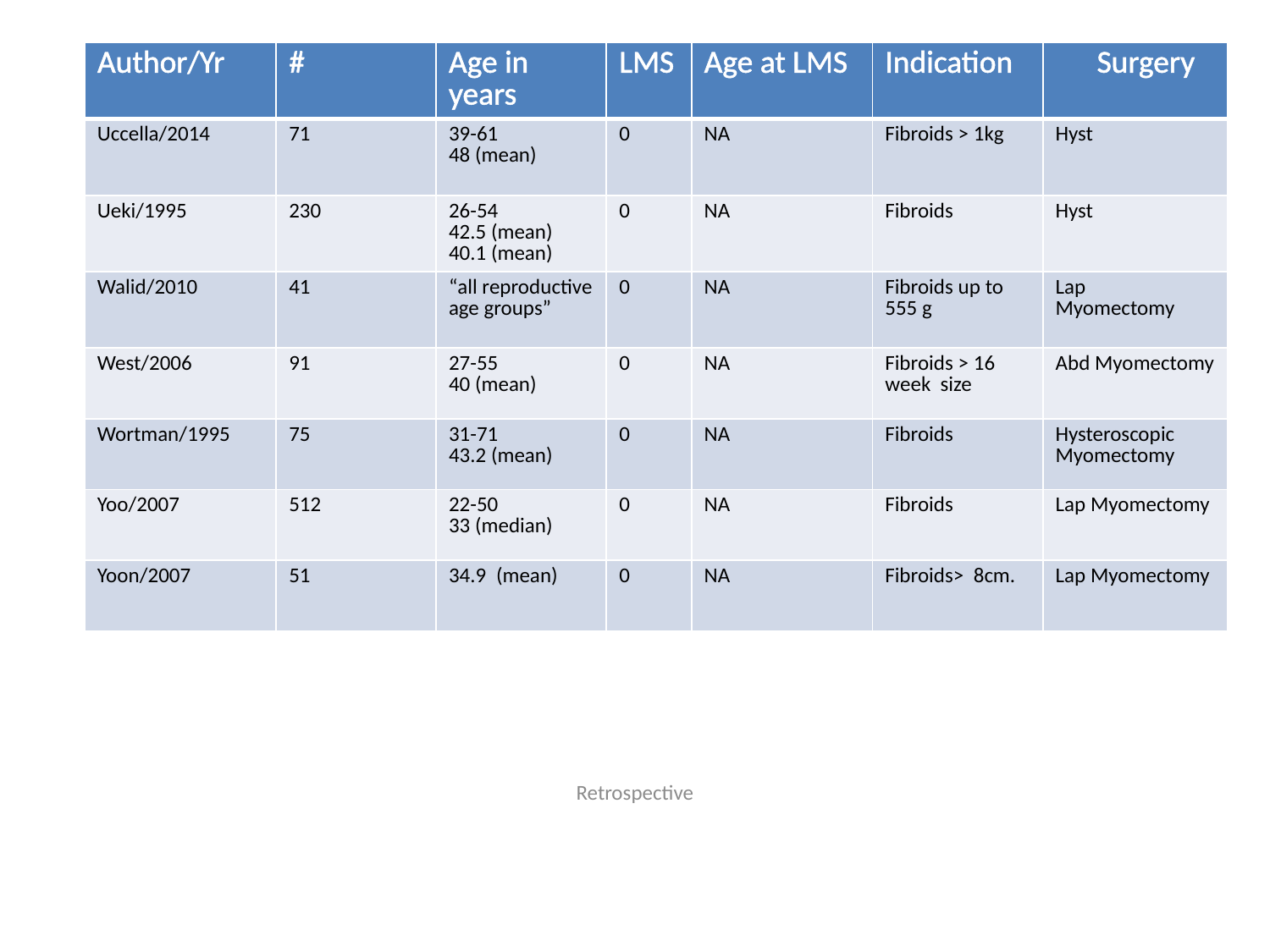

| Author/Yr | # | Age in years | LMS | Age at LMS | Indication | Surgery |
| --- | --- | --- | --- | --- | --- | --- |
| Uccella/2014 | 71 | 39-61 48 (mean) | 0 | NA | Fibroids > 1kg | Hyst |
| Ueki/1995 | 230 | 26-54 42.5 (mean) 40.1 (mean) | 0 | NA | Fibroids | Hyst |
| Walid/2010 | 41 | “all reproductive age groups” | 0 | NA | Fibroids up to 555 g | Lap Myomectomy |
| West/2006 | 91 | 27-55 40 (mean) | 0 | NA | Fibroids > 16 week size | Abd Myomectomy |
| Wortman/1995 | 75 | 31-71 43.2 (mean) | 0 | NA | Fibroids | Hysteroscopic Myomectomy |
| Yoo/2007 | 512 | 22-50 33 (median) | 0 | NA | Fibroids | Lap Myomectomy |
| Yoon/2007 | 51 | 34.9 (mean) | 0 | NA | Fibroids> 8cm. | Lap Myomectomy |
#
Retrospective

## Slide 12
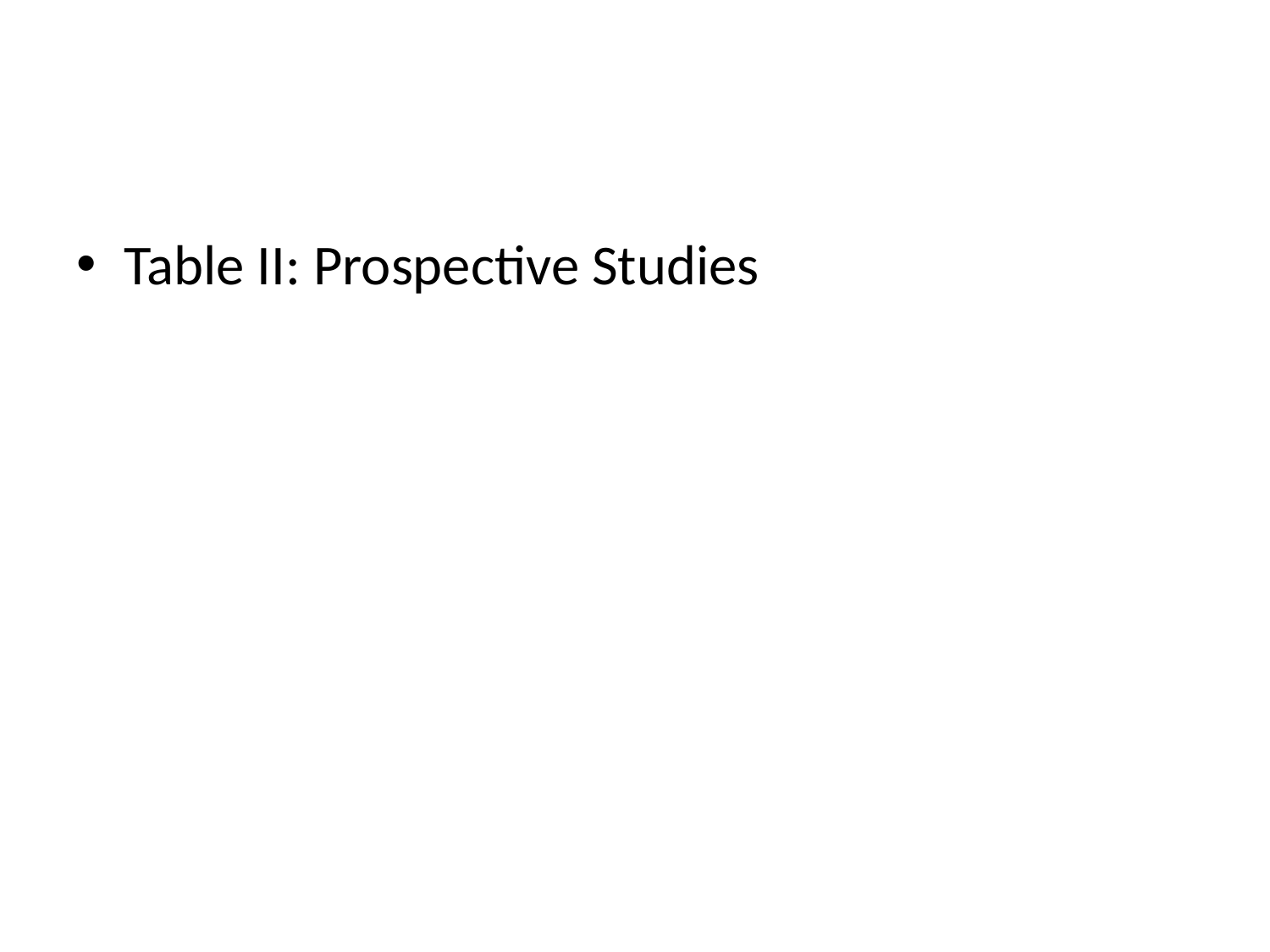

Table II: Prospective Studies

## Slide 13
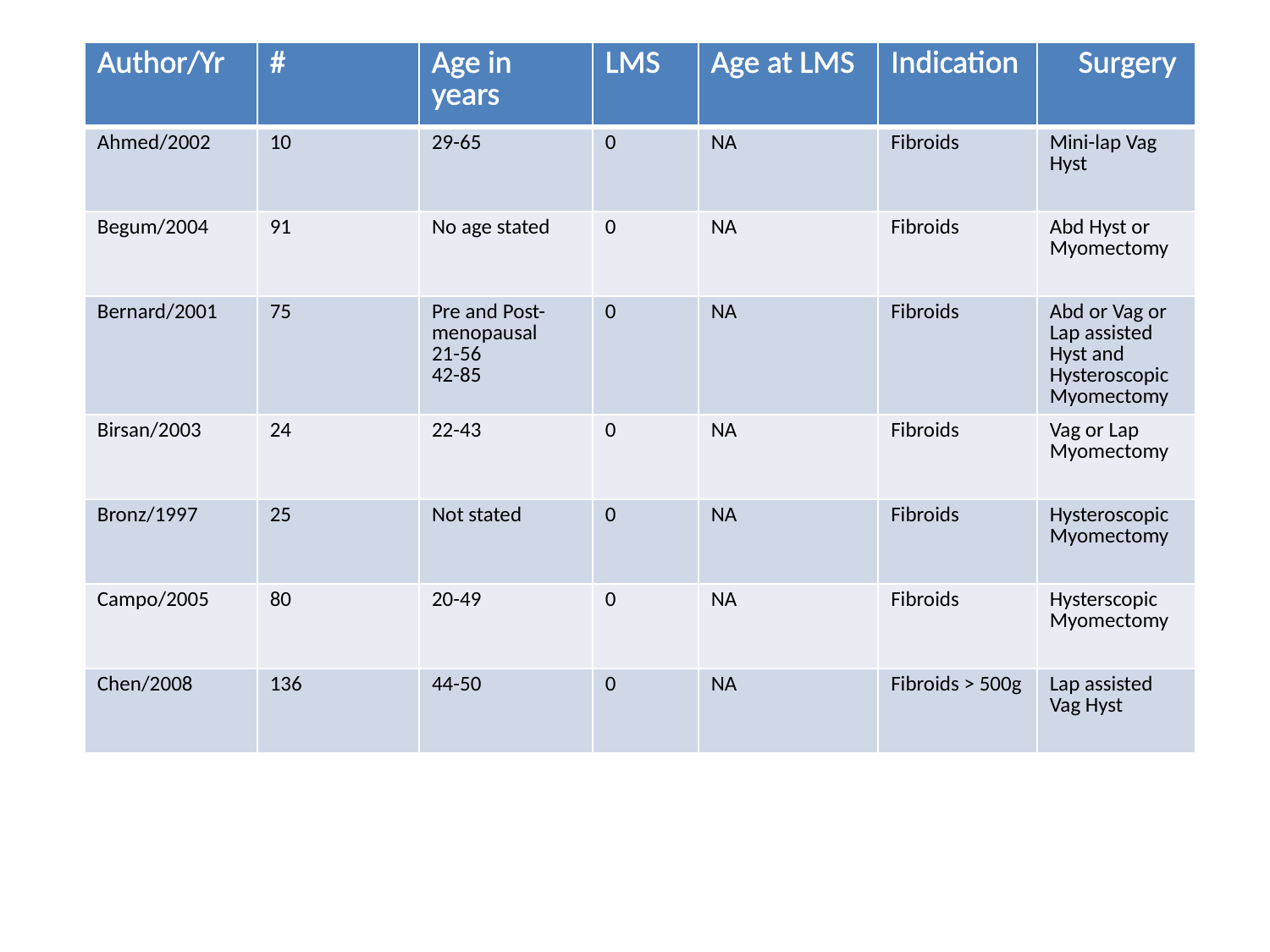

| Author/Yr | # | Age in years | LMS | Age at LMS | Indication | Surgery |
| --- | --- | --- | --- | --- | --- | --- |
| Ahmed/2002 | 10 | 29-65 | 0 | NA | Fibroids | Mini-lap Vag Hyst |
| Begum/2004 | 91 | No age stated | 0 | NA | Fibroids | Abd Hyst or Myomectomy |
| Bernard/2001 | 75 | Pre and Post-menopausal 21-56 42-85 | 0 | NA | Fibroids | Abd or Vag or Lap assisted Hyst and Hysteroscopic Myomectomy |
| Birsan/2003 | 24 | 22-43 | 0 | NA | Fibroids | Vag or Lap Myomectomy |
| Bronz/1997 | 25 | Not stated | 0 | NA | Fibroids | Hysteroscopic Myomectomy |
| Campo/2005 | 80 | 20-49 | 0 | NA | Fibroids | Hysterscopic Myomectomy |
| Chen/2008 | 136 | 44-50 | 0 | NA | Fibroids > 500g | Lap assisted Vag Hyst |
#

## Slide 14
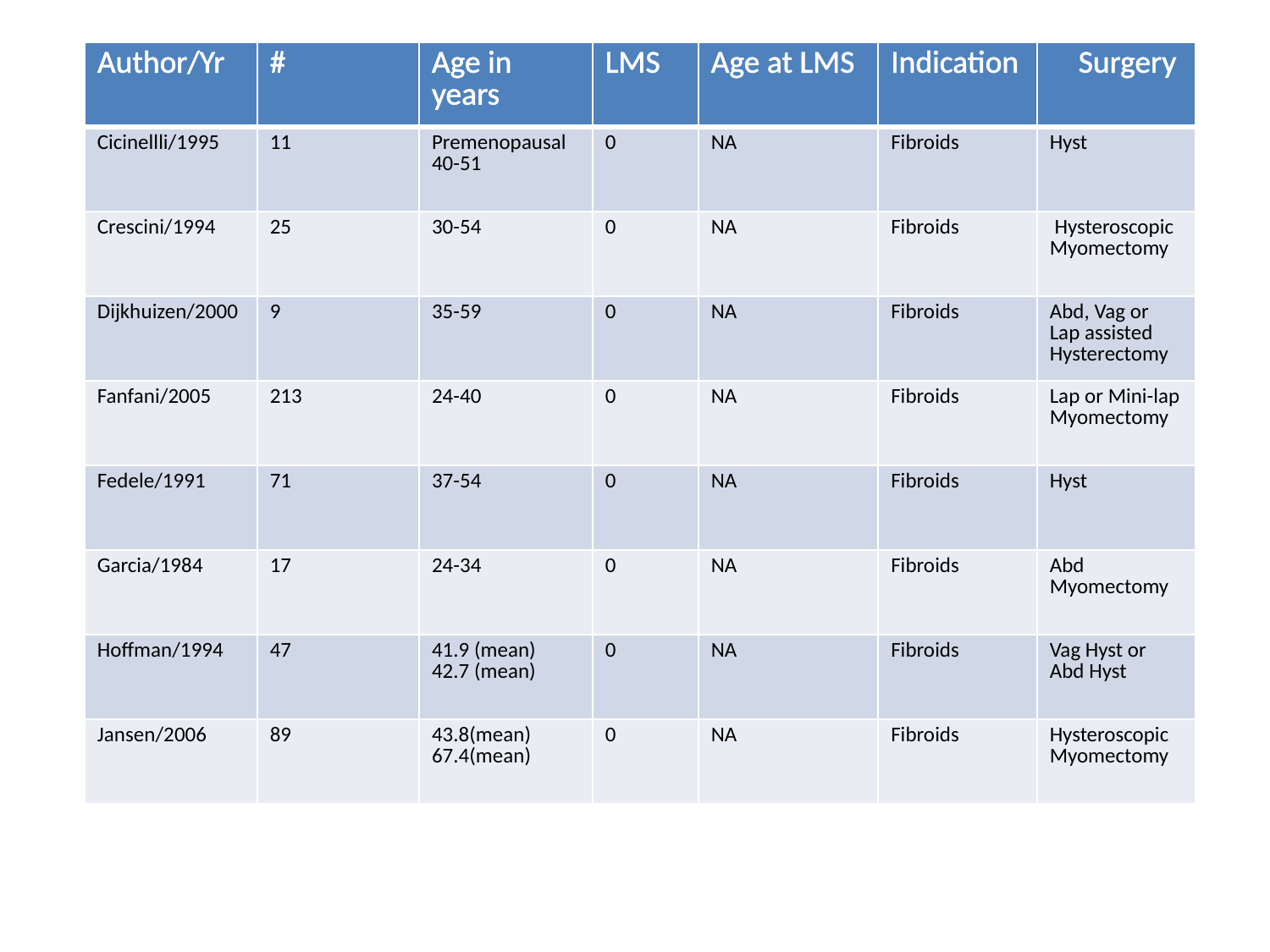

| Author/Yr | # | Age in years | LMS | Age at LMS | Indication | Surgery |
| --- | --- | --- | --- | --- | --- | --- |
| Cicinellli/1995 | 11 | Premenopausal 40-51 | 0 | NA | Fibroids | Hyst |
| Crescini/1994 | 25 | 30-54 | 0 | NA | Fibroids | Hysteroscopic Myomectomy |
| Dijkhuizen/2000 | 9 | 35-59 | 0 | NA | Fibroids | Abd, Vag or Lap assisted Hysterectomy |
| Fanfani/2005 | 213 | 24-40 | 0 | NA | Fibroids | Lap or Mini-lap Myomectomy |
| Fedele/1991 | 71 | 37-54 | 0 | NA | Fibroids | Hyst |
| Garcia/1984 | 17 | 24-34 | 0 | NA | Fibroids | Abd Myomectomy |
| Hoffman/1994 | 47 | 41.9 (mean) 42.7 (mean) | 0 | NA | Fibroids | Vag Hyst or Abd Hyst |
| Jansen/2006 | 89 | 43.8(mean) 67.4(mean) | 0 | NA | Fibroids | Hysteroscopic Myomectomy |
#

## Slide 15
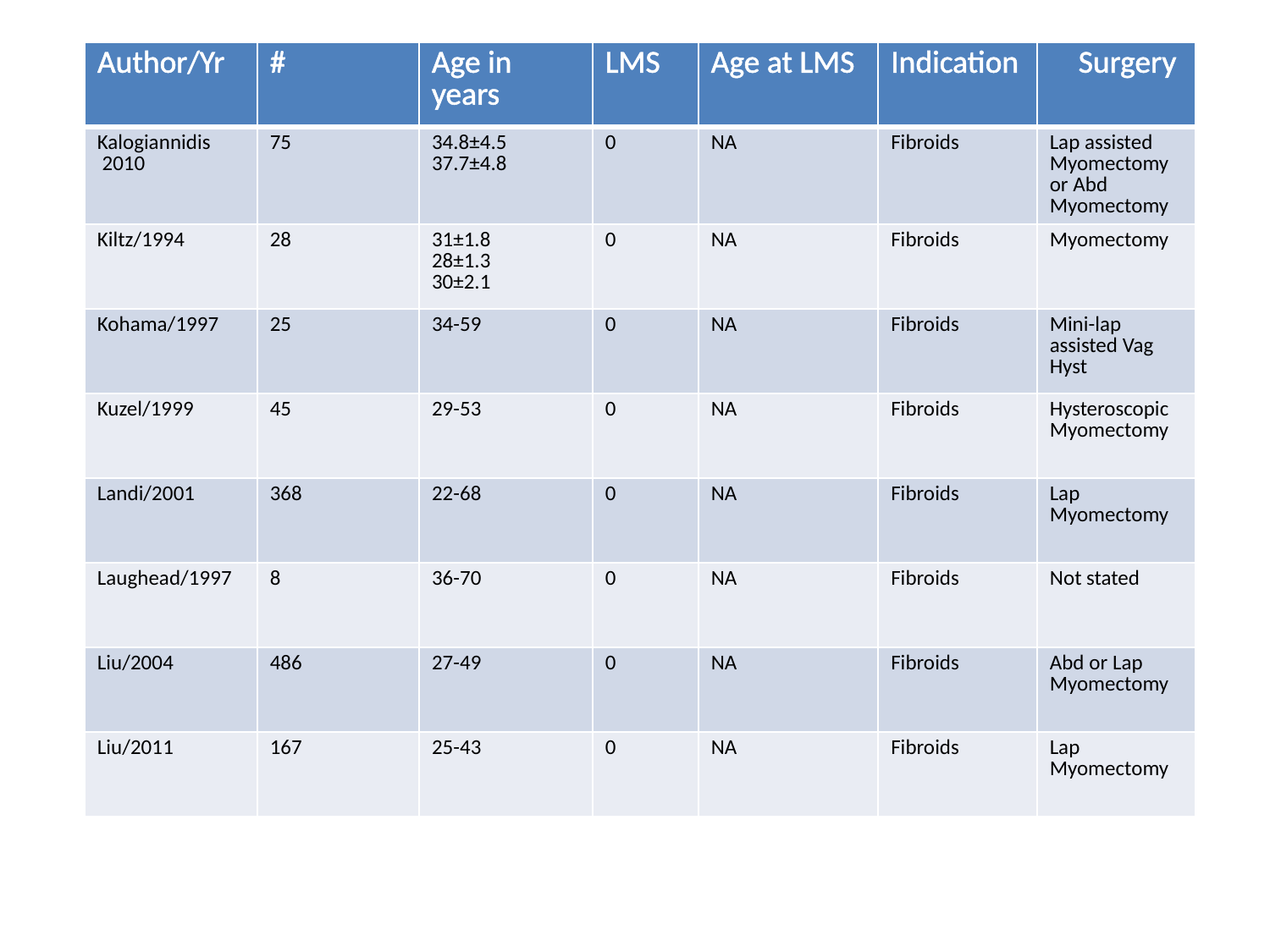

| Author/Yr | # | Age in years | LMS | Age at LMS | Indication | Surgery |
| --- | --- | --- | --- | --- | --- | --- |
| Kalogiannidis 2010 | 75 | 34.8±4.5 37.7±4.8 | 0 | NA | Fibroids | Lap assisted Myomectomy or Abd Myomectomy |
| Kiltz/1994 | 28 | 31±1.8 28±1.3 30±2.1 | 0 | NA | Fibroids | Myomectomy |
| Kohama/1997 | 25 | 34-59 | 0 | NA | Fibroids | Mini-lap assisted Vag Hyst |
| Kuzel/1999 | 45 | 29-53 | 0 | NA | Fibroids | Hysteroscopic Myomectomy |
| Landi/2001 | 368 | 22-68 | 0 | NA | Fibroids | Lap Myomectomy |
| Laughead/1997 | 8 | 36-70 | 0 | NA | Fibroids | Not stated |
| Liu/2004 | 486 | 27-49 | 0 | NA | Fibroids | Abd or Lap Myomectomy |
| Liu/2011 | 167 | 25-43 | 0 | NA | Fibroids | Lap Myomectomy |
#

## Slide 16
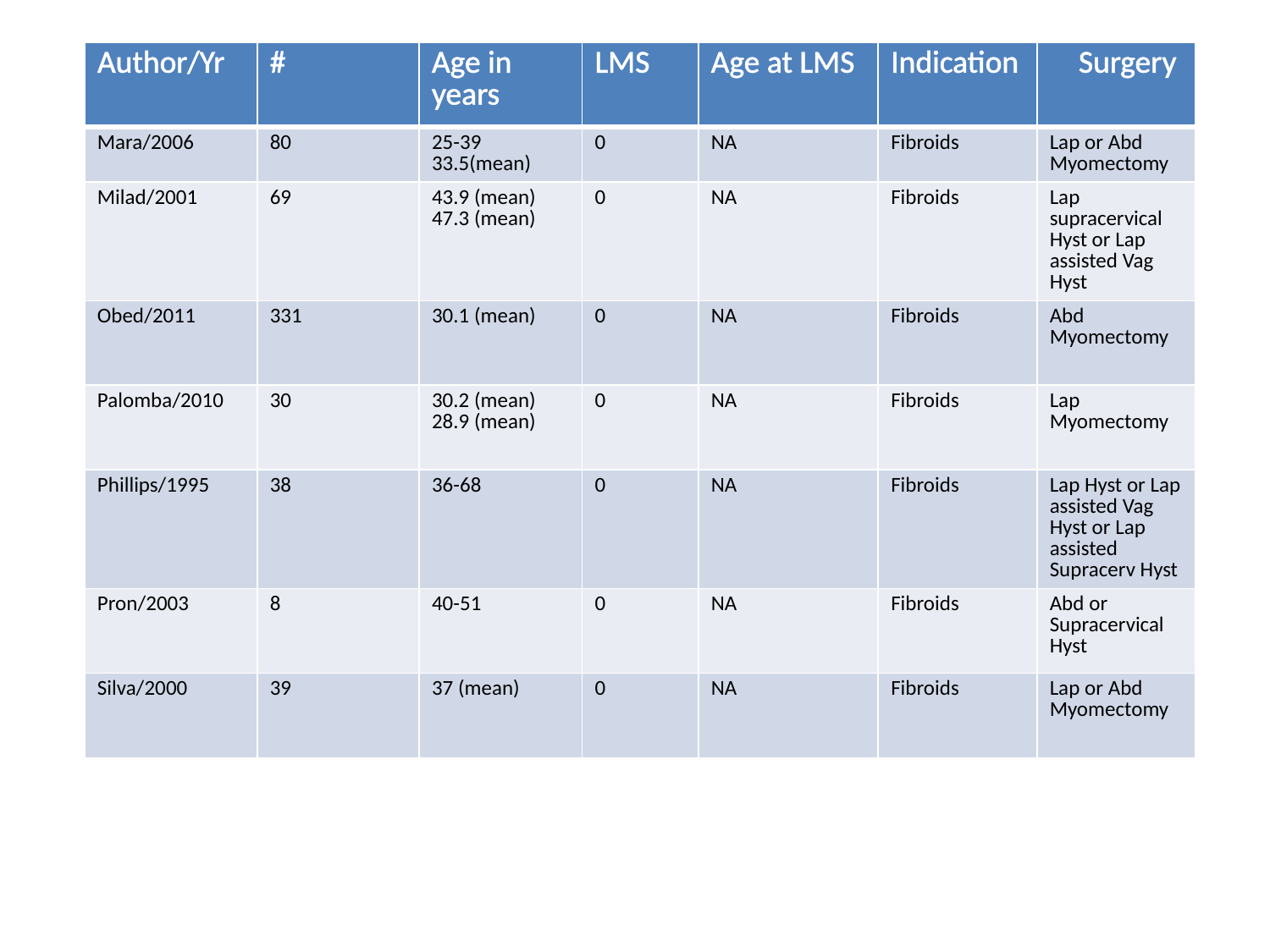

| Author/Yr | # | Age in years | LMS | Age at LMS | Indication | Surgery |
| --- | --- | --- | --- | --- | --- | --- |
| Mara/2006 | 80 | 25-39 33.5(mean) | 0 | NA | Fibroids | Lap or Abd Myomectomy |
| Milad/2001 | 69 | 43.9 (mean) 47.3 (mean) | 0 | NA | Fibroids | Lap supracervical Hyst or Lap assisted Vag Hyst |
| Obed/2011 | 331 | 30.1 (mean) | 0 | NA | Fibroids | Abd Myomectomy |
| Palomba/2010 | 30 | 30.2 (mean) 28.9 (mean) | 0 | NA | Fibroids | Lap Myomectomy |
| Phillips/1995 | 38 | 36-68 | 0 | NA | Fibroids | Lap Hyst or Lap assisted Vag Hyst or Lap assisted Supracerv Hyst |
| Pron/2003 | 8 | 40-51 | 0 | NA | Fibroids | Abd or Supracervical Hyst |
| Silva/2000 | 39 | 37 (mean) | 0 | NA | Fibroids | Lap or Abd Myomectomy |
#

## Slide 17
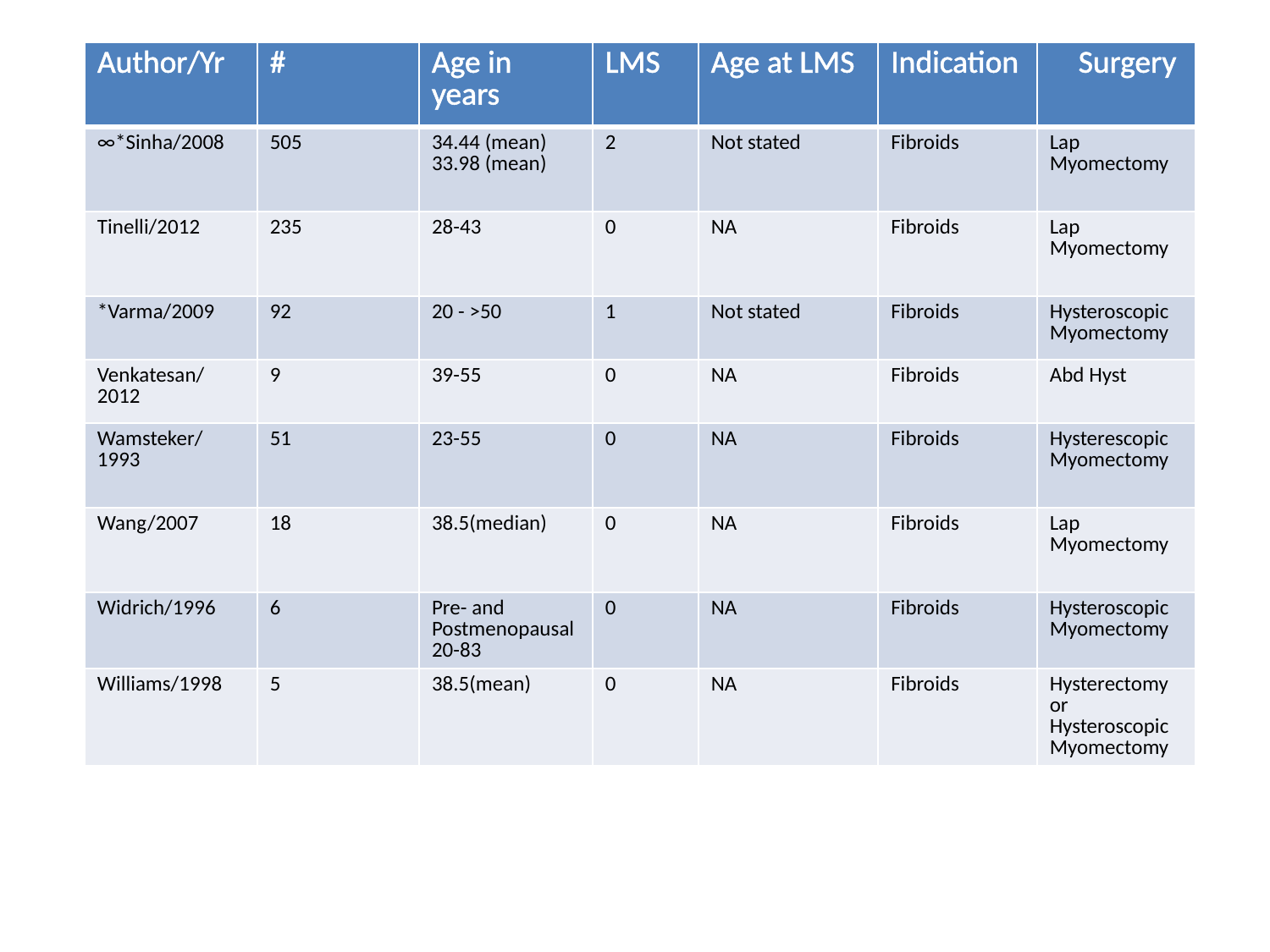

| Author/Yr | # | Age in years | LMS | Age at LMS | Indication | Surgery |
| --- | --- | --- | --- | --- | --- | --- |
| ∞\*Sinha/2008 | 505 | 34.44 (mean) 33.98 (mean) | 2 | Not stated | Fibroids | Lap Myomectomy |
| Tinelli/2012 | 235 | 28-43 | 0 | NA | Fibroids | Lap Myomectomy |
| \*Varma/2009 | 92 | 20 - >50 | 1 | Not stated | Fibroids | Hysteroscopic Myomectomy |
| Venkatesan/ 2012 | 9 | 39-55 | 0 | NA | Fibroids | Abd Hyst |
| Wamsteker/ 1993 | 51 | 23-55 | 0 | NA | Fibroids | Hysterescopic Myomectomy |
| Wang/2007 | 18 | 38.5(median) | 0 | NA | Fibroids | Lap Myomectomy |
| Widrich/1996 | 6 | Pre- and Postmenopausal20-83 | 0 | NA | Fibroids | Hysteroscopic Myomectomy |
| Williams/1998 | 5 | 38.5(mean) | 0 | NA | Fibroids | Hysterectomy or Hysteroscopic Myomectomy |
#

## Slide 18
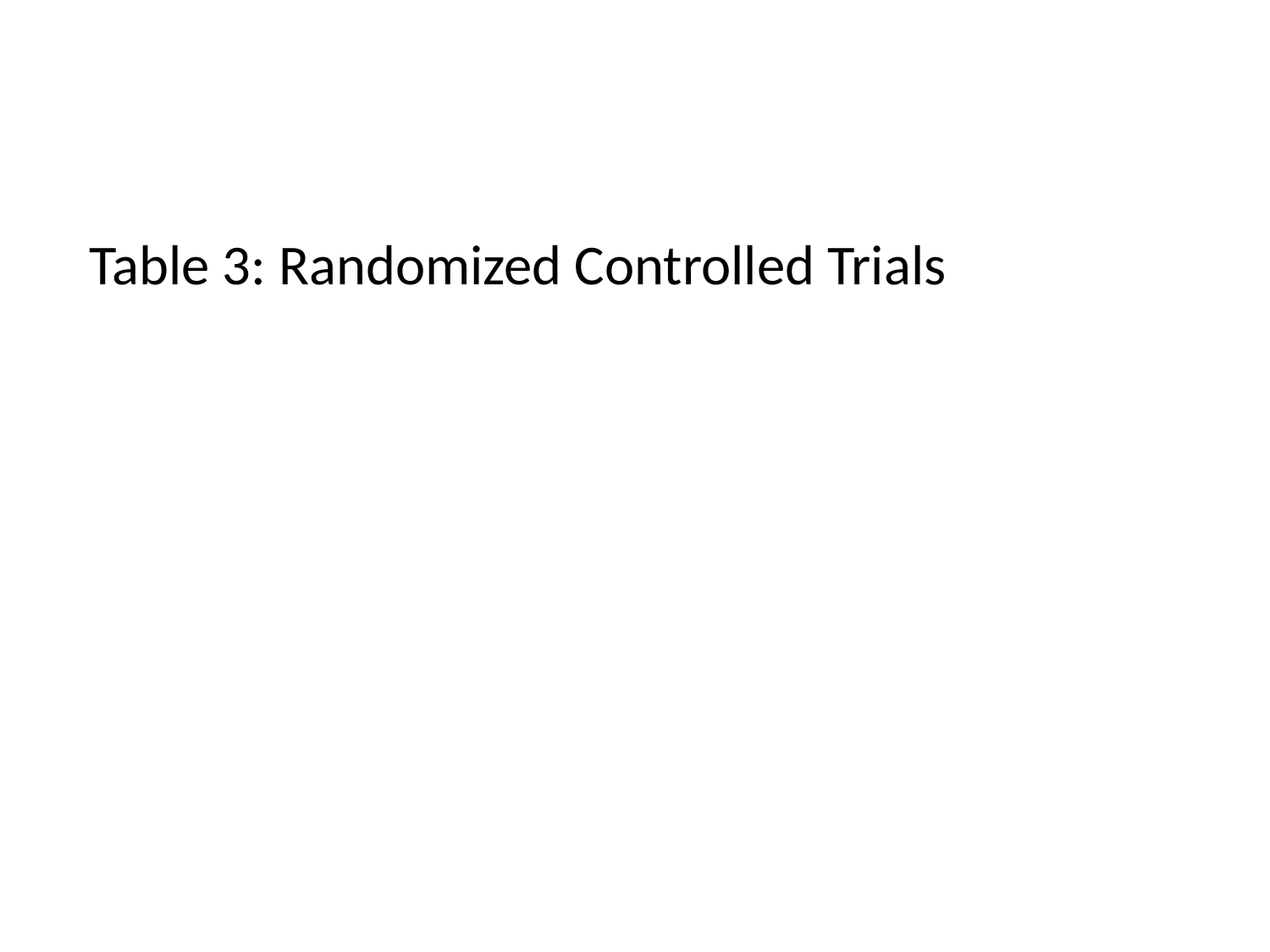

Table 3: Randomized Controlled Trials

## Slide 19
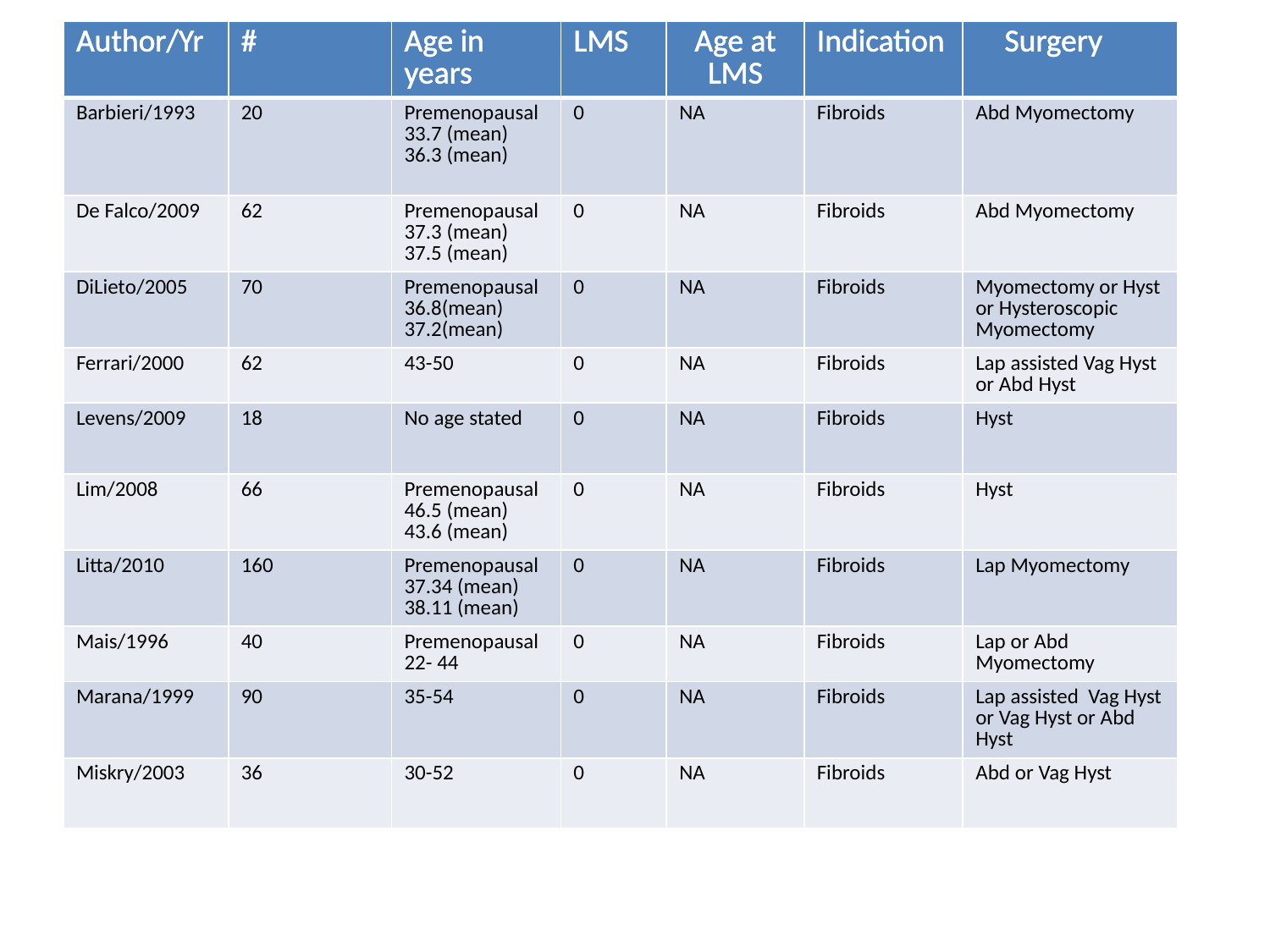

| Author/Yr | # | Age in years | LMS | Age at LMS | Indication | Surgery |
| --- | --- | --- | --- | --- | --- | --- |
| Barbieri/1993 | 20 | Premenopausal33.7 (mean) 36.3 (mean) | 0 | NA | Fibroids | Abd Myomectomy |
| De Falco/2009 | 62 | Premenopausal37.3 (mean) 37.5 (mean) | 0 | NA | Fibroids | Abd Myomectomy |
| DiLieto/2005 | 70 | Premenopausal36.8(mean) 37.2(mean) | 0 | NA | Fibroids | Myomectomy or Hyst or Hysteroscopic Myomectomy |
| Ferrari/2000 | 62 | 43-50 | 0 | NA | Fibroids | Lap assisted Vag Hyst or Abd Hyst |
| Levens/2009 | 18 | No age stated | 0 | NA | Fibroids | Hyst |
| Lim/2008 | 66 | Premenopausal46.5 (mean) 43.6 (mean) | 0 | NA | Fibroids | Hyst |
| Litta/2010 | 160 | Premenopausal37.34 (mean) 38.11 (mean) | 0 | NA | Fibroids | Lap Myomectomy |
| Mais/1996 | 40 | Premenopausal22- 44 | 0 | NA | Fibroids | Lap or Abd Myomectomy |
| Marana/1999 | 90 | 35-54 | 0 | NA | Fibroids | Lap assisted Vag Hyst or Vag Hyst or Abd Hyst |
| Miskry/2003 | 36 | 30-52 | 0 | NA | Fibroids | Abd or Vag Hyst |
#

## Slide 20
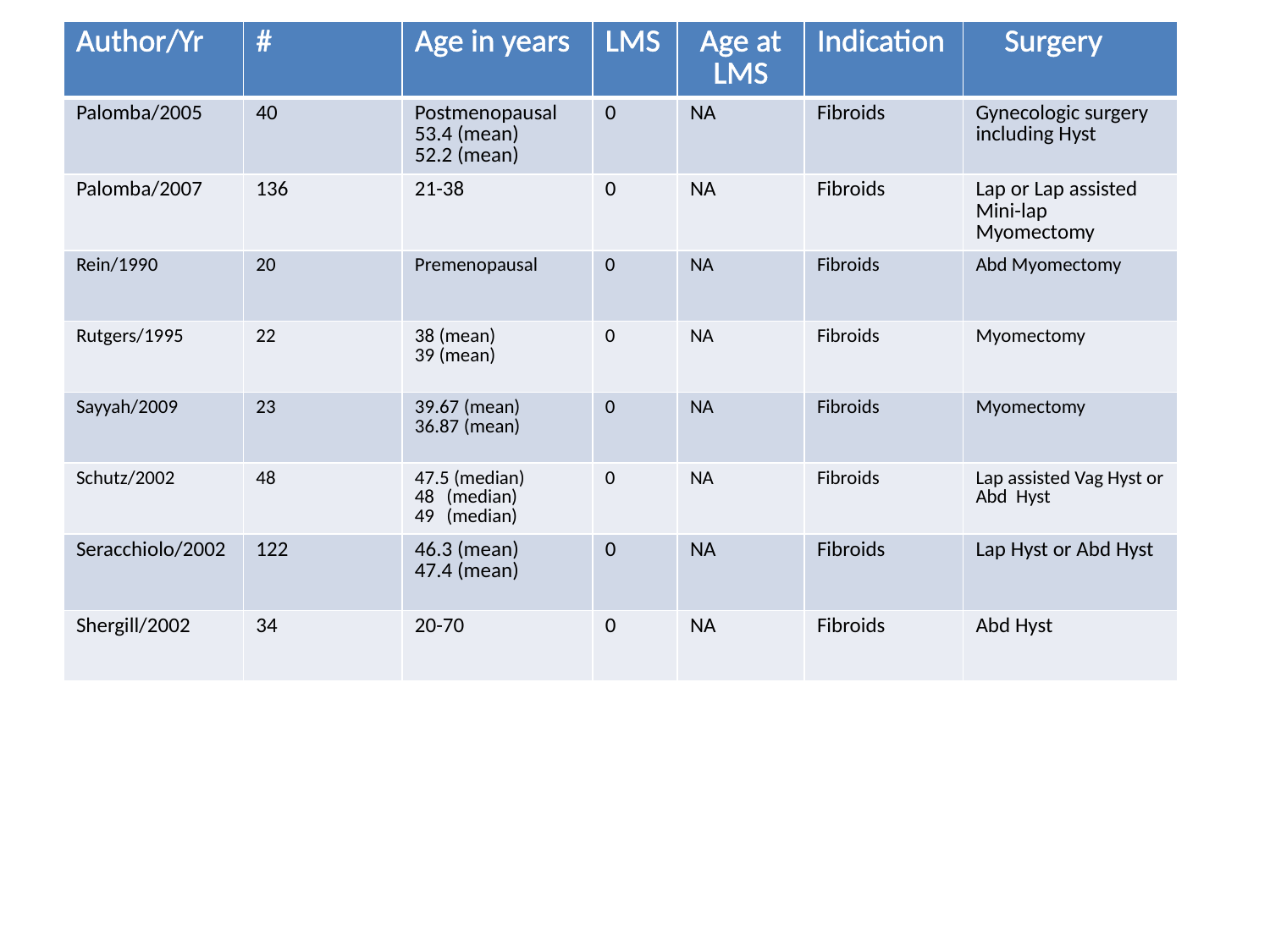

| Author/Yr | # | Age in years | LMS | Age at LMS | Indication | Surgery |
| --- | --- | --- | --- | --- | --- | --- |
| Palomba/2005 | 40 | Postmenopausal 53.4 (mean) 52.2 (mean) | 0 | NA | Fibroids | Gynecologic surgery including Hyst |
| Palomba/2007 | 136 | 21-38 | 0 | NA | Fibroids | Lap or Lap assisted Mini-lap Myomectomy |
| Rein/1990 | 20 | Premenopausal | 0 | NA | Fibroids | Abd Myomectomy |
| Rutgers/1995 | 22 | 38 (mean) 39 (mean) | 0 | NA | Fibroids | Myomectomy |
| Sayyah/2009 | 23 | 39.67 (mean) 36.87 (mean) | 0 | NA | Fibroids | Myomectomy |
| Schutz/2002 | 48 | 47.5 (median) (median) (median) | 0 | NA | Fibroids | Lap assisted Vag Hyst or Abd Hyst |
| Seracchiolo/2002 | 122 | 46.3 (mean) 47.4 (mean) | 0 | NA | Fibroids | Lap Hyst or Abd Hyst |
| Shergill/2002 | 34 | 20-70 | 0 | NA | Fibroids | Abd Hyst |
# 4.9

## Slide 21
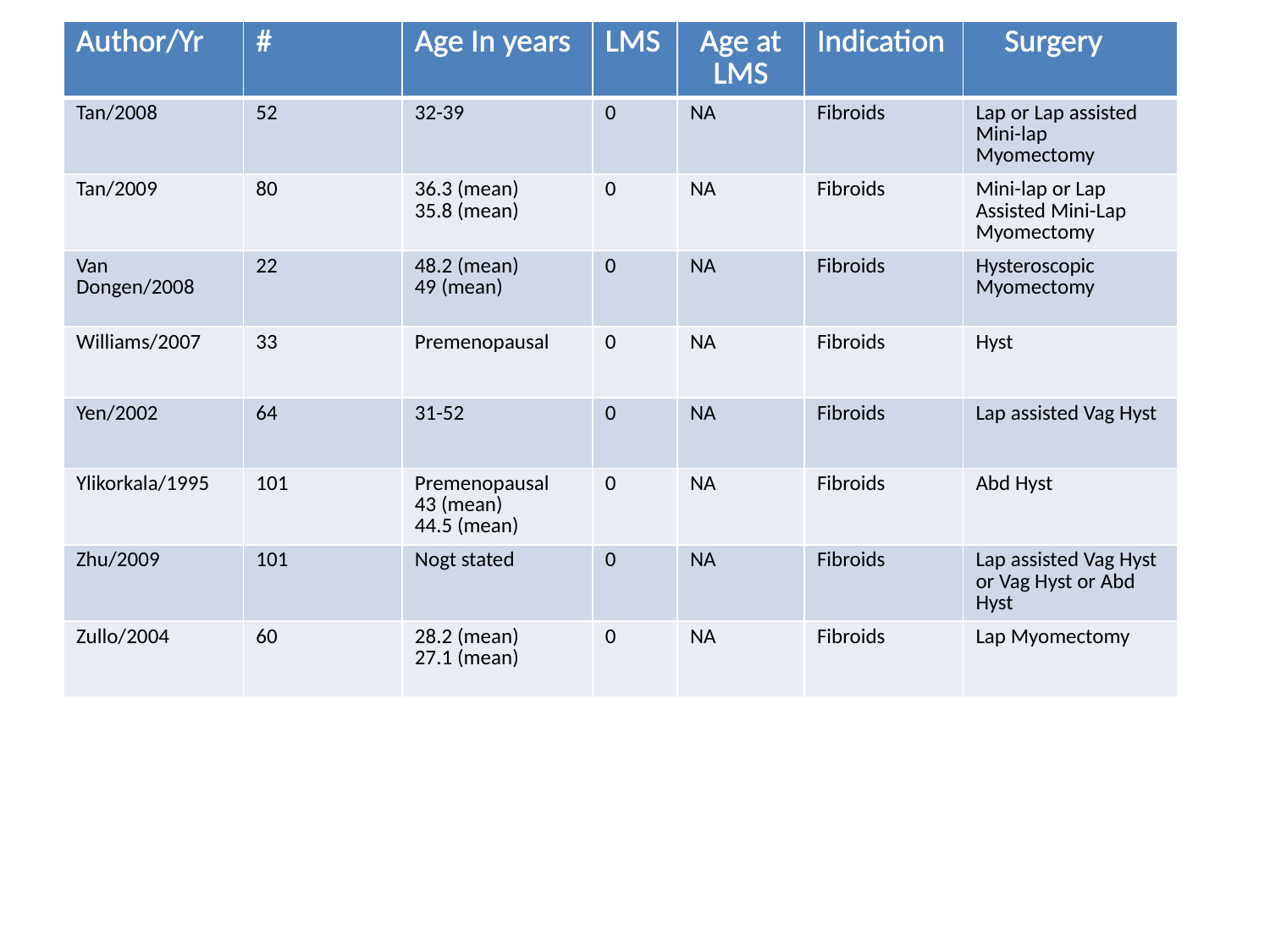

| Author/Yr | # | Age In years | LMS | Age at LMS | Indication | Surgery |
| --- | --- | --- | --- | --- | --- | --- |
| Tan/2008 | 52 | 32-39 | 0 | NA | Fibroids | Lap or Lap assisted Mini-lap Myomectomy |
| Tan/2009 | 80 | 36.3 (mean) 35.8 (mean) | 0 | NA | Fibroids | Mini-lap or Lap Assisted Mini-Lap Myomectomy |
| Van Dongen/2008 | 22 | 48.2 (mean) 49 (mean) | 0 | NA | Fibroids | Hysteroscopic Myomectomy |
| Williams/2007 | 33 | Premenopausal | 0 | NA | Fibroids | Hyst |
| Yen/2002 | 64 | 31-52 | 0 | NA | Fibroids | Lap assisted Vag Hyst |
| Ylikorkala/1995 | 101 | Premenopausal 43 (mean) 44.5 (mean) | 0 | NA | Fibroids | Abd Hyst |
| Zhu/2009 | 101 | Nogt stated | 0 | NA | Fibroids | Lap assisted Vag Hyst or Vag Hyst or Abd Hyst |
| Zullo/2004 | 60 | 28.2 (mean) 27.1 (mean) | 0 | NA | Fibroids | Lap Myomectomy |
# 4.9
