# Supplementary material for: The prevalence of occult leiomyosarcoma at surgery for presumed uterine fibroids: a meta-analysis
Source: Gynecol Surg. 2015 May 19;12(3):165–77. doi: 10.1007/s10397-015-0894-4 (PMC4532723; doi:10.1007/s10397-015-0894-4)
Supplement: Supplementary file 2 — A detailed description of the Bayesian model used in this analysis, including the code used for running the data. (DOCX 40 kb) [file 10397_2015_894_MOESM2_ESM.docx]

The prevalence of occult leiomyosarcoma at surgery for presumed uterine fibroids: A meta-analysis
Elizabeth A. Pritts, MD^1^, David J. Vanness, PhD^2^, Jonathan S. Berek, MD, MMS^3^, William Parker, MD^4^, Ronald Feinberg, MD, PhD^5^, Jacqueline Feinberg, BA^5^, David L. Olive, MD^1^.
 
^1^Wisconsin Fertility Institute, ^2^University of Wisconsin School of Medicine and Public Health, ^3^Stanford University School of Medicine, ^4^University of California, Los Angeles,^5^Reproductive Associates of Delaware.
 
Corresponding Author: Elizabeth Pritts, MD. Email: epritts@wisconsinfertilty.com

Supplemental Digital Content 2: Bayesian statistical details and model code

We conducted meta-analysis using a Bayesian random effects binomial-logit likelihood model specified as follows:

|  | $r_{i} \sim\text{Binomial}\left( p_{i}, n_{i} \right)$ | 1a. |
| --- | --- | --- |
|  | $\log\left( \frac{p_{i}}{1-p\left( i \right)} \right)= \alpha_{i}$ | 1b. |
|  | $\alpha_{i} \sim N\left( \mu_{\alpha},\sigma^{2} \right)$ | 1c. |

where $r_{i}$ is the observed number of leiomyosarcomas out of $n_{i}$ cases observed in study $i$. Random effects models assume exchangeability; that is, the prevalence of leiomyosarcoma is assumed to vary from study to study around an overall population mean rate for reasons other than sampling variability, but it is not possible to rank-order studies by their predicted rates *a priori*. The log-odds of leiomyosarcoma for each study, $\log\left( \frac{p_{i}}{1-p\left( i \right)} \right)$, are assumed to be distributed normally around the mean log-odds, $\mu_{a}$, with between-study heterogeneity $\sigma^{2}$).

Transforming from the log-odds to the probability scale, predicted prevalence of leiomyosarcoma is therefore:

|  | $p^{*}= \frac{e^{\mu_{a}}}{1+e^{\mu_{a}}}$ | 2. |
| --- | --- | --- |

We used a minimally informative normal prior for the mean of the random-effects distribution and a wide uniform prior for between-study heterogeneity:

|  | $\mu_{a} \sim N\left( 0,10000 \right)$ | 3a. |
| --- | --- | --- |
|  | $\sigma\sim U(0, 10)$ | 3b. |

Separate analyses were conducted for prospective studies only ($n=64$), retrospective studies only ($n=70$) and both types combined ($n=134$). For sensitivity analysis, we applied our Bayesian specification to the following data: 1) reassigning seven tumors from 3 retrospective studies not meeting criteria for leiomyosarcoma to be non-events (combined); 2) excluding studies with $n < 100$ (prospective and combined); 3) add one leiomyosarcoma to the trial with the largest sample size and no reported events (prospective and combined); 4) add one leiomyosarcoma to the trial with the smallest sample size and no reported events (prospective and combined); 5) reallocating all leiomyosarcomas to studies based on sample size to investigate the effect of heterogeneity in observed rates (combined); 6) dataset used by the FDA in its meta-analysis ($n=9$).

We used Markov Chain Monte Carlo (MCMC) simulation to estimate the posterior distribution of model parameters and predicted leiomyosarcoma prevalence $p^{*}$ using JAGS (v. 3.3) in the R statistical environment (v. 3.1.1 for 64 bit Windows) with packages rjags (v. 3-13) and coda (v. 0.16-1). JAGS model code is provided in Figure S. Parameter and prevalence point estimates were derived from the posterior mean, and 95% credible intervals (CrI) were constructed from the posterior .025 and .975 percentiles. Unlike confidence intervals (CI) generated from classical estimation procedures, it is permissible to interpret the 95% credible interval as having 95% probability of containing the true unknown prevalence, given the available data and model specification.

Ten parallel chains were run in adaptive mode for 100,000 iterations. Inference samples were then drawn from an additional 500,000 iterations per chain, thinned 100 to 1 and pooled to yield 50,000 total posterior samples. Convergence was confirmed using the Gelman-Rubin diagnostic, which indicated a ratio of between-chain to within-chain variance (potential scale reduction factor) of less or equal to 1.01 for all model parameters in the base case and all sensitivity, suggesting that all ten chains were sampling from the same posterior distribution.

Full posterior summaries are provided in Table S0 for the base case analyses and in Tables S1-S6 for the sensitivity analyses.

Table S0. Full Markov Chain Monte Carlo Estimation Results: Base Case

| Base Case All Studies (n=134) | | |  |  |  |  |  |  |  |  |
| --- | --- | --- | --- | --- | --- | --- | --- | --- | --- | --- |
|  | Posterior Mean | Posterior Standard Deviation | Naïve Standard Error | Time Series Standard Error | Posterior 2.5%ile | Posterior 25%ile | Posterior 50%ile | Posterior 75%ile | Posterior 97.5%ile | Potential Scale Reduction Factor |
| μ_α_ | -7.68460 | 0.46618 | 0.00209 | 0.00271 | -8.75520 | -7.95195 | -7.63135 | -7.35643 | -6.93001 | 1 |
| p* | 0.00051 | 0.00021 | 0.00000 | 0.00000 | 0.00016 | 0.00035 | 0.00048 | 0.00064 | 0.00098 | 1 |
| σ | 1.11450 | 0.36305 | 0.00162 | 0.00221 | 0.50386 | 0.86426 | 1.08071 | 1.32867 | 1.92598 | 1 |
| Penalized | deviance: | 99.11 |  |  |  |  |  |  |  |  |
|  |  |  |  |  |  |  |  |  |  |  |
| Base Case Prospective studies (n=64) | | | |  |  |  |  |  |  |  |
|  | Posterior Mean | Posterior Standard Deviation | Naïve Standard Error | Time Series Standard Error | Posterior 2.5%ile | Posterior 25%ile | Posterior 50%ile | Posterior 75%ile | Posterior 97.5%ile | Potential Scale Reduction Factor |
| μ_α_ | -12.62000 | 4.84621 | 0.02167 | 0.05518 | -24.63000 | -15.29000 | -11.08000 | -8.83167 | -7.18860 | 1 |
| p* | 0.00012 | 0.00022 | 0.00000 | 0.00000 | 0.00000 | 0.00000 | 0.00002 | 0.00015 | 0.00075 | 1 |
| σ | 3.52800 | 2.53730 | 0.01135 | 0.03052 | 0.19030 | 1.51000 | 2.88900 | 5.16112 | 9.30826 | 1 |
| Penalized | deviance: | 22.25 |  |  |  |  |  |  |  |  |
|  |  |  |  |  |  |  |  |  |  |  |
| Base Case Retrospective Studies (n=70) | | | |  |  |  |  |  |  |  |
|  | Posterior Mean | Posterior Standard Deviation | Naïve Standard Error | Time Series Standard Error | Posterior 2.5%ile | Posterior 25%ile | Posterior 50%ile | Posterior 75%ile | Posterior 97.5%ile | Potential Scale Reduction Factor |
| μ_α_ | -7.56974 | 0.49201 | 0.00220 | 0.00245 | -8.70212 | -7.84848 | -7.50929 | -7.22432 | -6.78076 | 1 |
| p* | 0.00057 | 0.00025 | 0.00000 | 0.00000 | 0.00017 | 0.00039 | 0.00055 | 0.00073 | 0.00113 | 1 |
| σ | 1.14562 | 0.41092 | 0.00184 | 0.00213 | 0.46701 | 0.86173 | 1.09951 | 1.38237 | 2.07793 | 1 |
| Penalized | deviance: | 80.76 |  |  |  |  |  |  |  |  |

Table S1. Full Markov Chain Monte Carlo Estimation Results: Sensitivity Analysis 1 - Recategorizing Seven Non-Leiomyosarcomas

| Recategorizing Seven Non-Leiomyosarcomas (n=134) | | | | |  |  |  |  |  |  |
| --- | --- | --- | --- | --- | --- | --- | --- | --- | --- | --- |
|  | Posterior Mean | Posterior Standard Deviation | Naïve Standard Error | Time Series Standard Error | Posterior 2.5%ile | Posterior 25%ile | Posterior 50%ile | Posterior 75%ile | Posterior 97.5%ile | Potential Scale Reduction Factor |
| μ_α_ | -7.63550 | 0.47310 | 0.00212 | 0.00396 | -8.74090 | -7.89562 | -7.56692 | -7.29684 | -6.92004 | 1 |
| p* | 0.00053 | 0.00022 | 0.00000 | 0.00000 | 0.00016 | 0.00037 | 0.00052 | 0.00068 | 0.00099 | 1 |
| σ | 0.88898 | 0.44242 | 0.00198 | 0.00450 | 0.09794 | 0.58103 | 0.86685 | 1.16460 | 1.83391 | 1 |
| Penalized | deviance: | 94.64 |  |  |  |  |  |  |  |  |

Table S2. Full Markov Chain Monte Carlo Estimation Results: Sensitivity Analysis 2 – Exclude Studies with n<100

| Studies with N ≥ 100 (Combined; n=57) | | | |  |  |  |  |  |  |  |
| --- | --- | --- | --- | --- | --- | --- | --- | --- | --- | --- |
|  | Posterior Mean | Posterior Standard Deviation | Naïve Standard Error | Time Series Standard Error | Posterior 2.5%ile | Posterior 25%ile | Posterior 50%ile | Posterior 75%ile | Posterior 97.5%ile | Potential Scale Reduction Factor |
| μ_α_ | -7.60732 | 0.46934 | 0.00210 | 0.00223 | -8.68421 | -7.87391 | -7.55121 | -7.27908 | -6.85129 | 1 |
| p* | 0.00055 | 0.00023 | 0.00000 | 0.00000 | 0.00017 | 0.00038 | 0.00053 | 0.00069 | 0.00106 | 1 |
| σ | 1.06087 | 0.38180 | 0.00171 | 0.00189 | 0.44271 | 0.79990 | 1.01563 | 1.27304 | 1.93258 | 1 |
| Penalized | deviance: | 78.94 |  |  |  |  |  |  |  |  |
|  |  |  |  |  |  |  |  |  |  |  |
| Studies with N ≥ 100 (Prospective; n=13) | | | |  |  |  |  |  |  |  |
|  | Posterior Mean | Posterior Standard Deviation | Naïve Standard Error | Time Series Standard Error | Posterior 2.5%ile | Posterior 25%ile | Posterior 50%ile | Posterior 75%ile | Posterior 97.5%ile | Potential Scale Reduction Factor |
| μ_α_ | -15.22000 | 5.57010 | 0.02491 | 0.02708 | -27.93000 | -18.64000 | -14.35000 | -10.83000 | -7.39170 | 1 |
| p* | 0.00006 | 0.00020 | 0.00000 | 0.00000 | 0.00000 | 0.00000 | 0.00000 | 0.00002 | 0.00062 | 1 |
| σ | 5.49600 | 2.69384 | 0.01205 | 0.01367 | 0.61480 | 3.29000 | 5.60000 | 7.81000 | 9.78437 | 1 |
| Penalized | deviance: | 10.02 |  |  |  |  |  |  |  |  |

Table S3. Full Markov Chain Monte Carlo Estimation Results: Sensitivity Analysis 3 – Add One Leiomyosarcoma to the Largest Study with Zero Events

| (Combined;n=134) | | | | | | | |  |  |  |
| --- | --- | --- | --- | --- | --- | --- | --- | --- | --- | --- |
|  | Posterior Mean | Posterior Standard Deviation | Naïve Standard Error | Time Series Standard Error | Posterior 2.5%ile | Posterior 25%ile | Posterior 50%ile | Posterior 75%ile | Posterior 97.5%ile | Potential Scale Reduction Factor |
| μ_α_ | -7.52172 | 0.42138 | 0.00188 | 0.00270 | -8.46691 | -7.76881 | -7.47954 | -7.22352 | -6.82782 | 1 |
| p* | 0.00059 | 0.00023 | 0.00000 | 0.00000 | 0.00021 | 0.00042 | 0.00056 | 0.00073 | 0.00108 | 1 |
| σ | 0.98965 | 0.34164 | 0.00153 | 0.00261 | 0.38495 | 0.75929 | 0.96504 | 1.19392 | 1.73851 | 1 |
| Penalized | deviance: | 101.3 |  |  |  |  |  |  |  |  |

| (Prospective; n=64) | | | | | | | | | |  |
| --- | --- | --- | --- | --- | --- | --- | --- | --- | --- | --- |
|  | Posterior Mean | Posterior Standard Deviation | Naïve Standard Error | Time Series Standard Error | Posterior 2.5%ile | Posterior 25%ile | Posterior 50%ile | Posterior 75%ile | Posterior 97.5%ile | Potential Scale Reduction Factor |
| μ_α_ | -9.04219 | 2.55645 | 0.01143 | 0.03491 | -16.74000 | -9.58700 | -8.25002 | -7.50668 | -6.66984 | 1 |
| p* | 0.00036 | 0.00036 | 0.00000 | 0.00000 | 0.00000 | 0.00007 | 0.00026 | 0.00055 | 0.00127 | 1 |
| σ | 1.67692 | 1.59114 | 0.00712 | 0.02186 | 0.06665 | 0.60820 | 1.22481 | 2.17672 | 6.38947 | 1 |
| Penalized | deviance: | 24.94 |  |  |  |  |  |  |  |  |

Table S4. Full Markov Chain Monte Carlo Estimation Results: Sensitivity Analysis 4 – Add One Leiomyosarcoma to the Smallest Study with Zero Events

| (Combined;n=134) | | | | | | | |  |  |  |
| --- | --- | --- | --- | --- | --- | --- | --- | --- | --- | --- |
|  | Posterior Mean | Posterior Standard Deviation | Naïve Standard Error | Time Series Standard Error | Posterior 2.5%ile | Posterior 25%ile | Posterior 50%ile | Posterior 75%ile | Posterior 97.5%ile | Potential Scale Reduction Factor |
| μ_α_ | -7.63250 | 0.47022 | 0.00210 | 0.00273 | -8.71249 | -7.89497 | -7.57526 | -7.30462 | -6.88308 | 1 |
| p* | 0.00053 | 0.00022 | 0.00000 | 0.00000 | 0.00016 | 0.00037 | 0.00051 | 0.00067 | 0.00102 | 1 |
| σ | 1.13278 | 0.38567 | 0.00173 | 0.00233 | 0.49508 | 0.86673 | 1.09122 | 1.35473 | 2.01298 | 1 |
| Penalized | deviance: | 110.2 |  |  |  |  |  |  |  |  |

| (Prospective; n=64) | | | | | | | | | |  |
| --- | --- | --- | --- | --- | --- | --- | --- | --- | --- | --- |
|  | Posterior Mean | Posterior Standard Deviation | Naïve Standard Error | Time Series Standard Error | Posterior 2.5%ile | Posterior 25%ile | Posterior 50%ile | Posterior 75%ile | Posterior 97.5%ile | Potential Scale Reduction Factor |
| μ_α_ | -12.45000 | 4.35985 | 0.01950 | 0.04367 | -22.63000 | -15.18000 | -11.40000 | -8.89582 | -7.02664 | 1 |
| p* | 0.00013 | 0.00025 | 0.00000 | 0.00000 | 0.00000 | 0.00000 | 0.00001 | 0.00014 | 0.00089 | 1.01 |
| σ | 4.03100 | 2.54871 | 0.01140 | 0.02696 | 0.26940 | 1.97500 | 3.62900 | 5.82185 | 9.40576 | 1 |
| Penalized | deviance: | 32 |  |  |  |  |  |  |  |  |

Table S5. Full Markov Chain Monte Carlo Estimation Results: Sensitivity Analysis 5 – Reallocate 32 Leiomyosarcomas to Largest Studies (Heterogeneity Test)

| (Combined n=134) | | | | | | | | |  |  |
| --- | --- | --- | --- | --- | --- | --- | --- | --- | --- | --- |
|  | Posterior Mean | Posterior Standard Deviation | Naïve Standard Error | Time Series Standard Error | Posterior 2.5%ile | Posterior 25%ile | Posterior 50%ile | Posterior 75%ile | Posterior 97.5%ile | Potential Scale Reduction Factor |
| μ_α_ | -6.89556 | 0.18542 | 0.00083 | 0.00242 | -7.27382 | -7.01630 | -6.88989 | -6.76785 | -6.55023 | 1 |
| p* | 0.00103 | 0.00019 | 0.00000 | 0.00000 | 0.00069 | 0.00090 | 0.00102 | 0.00115 | 0.00143 | 1 |
| σ | 0.17059 | 0.13051 | 0.00058 | 0.00167 | 0.00712 | 0.06922 | 0.14267 | 0.24290 | 0.48929 | 1 |
| Penalized | deviance: | 85.77 |  |  |  |  |  |  |  |  |

Table S6. Full Markov Chain Monte Carlo Estimation Results: Sensitivity Analysis 6 – FDA Dataset

| (FDA Dataset; n=9) | |  |  |  |  |  |  |  |  |  |
| --- | --- | --- | --- | --- | --- | --- | --- | --- | --- | --- |
|  | Posterior Mean | Posterior Standard Deviation | Naïve Standard Error | Time Series Standard Error | Posterior 2.5%ile | Posterior 25%ile | Posterior 50%ile | Posterior 75%ile | Posterior 97.5%ile | Potential Scale Reduction Factor |
| μ_α_ | -6.35759 | 0.39336 | 0.00176 | 0.00179 | -7.25806 | -6.56748 | -6.31810 | -6.10228 | -5.70490 | 1 |
| p* | 0.00186 | 0.00074 | 0.00000 | 0.00000 | 0.00070 | 0.00140 | 0.00180 | 0.00223 | 0.00332 | 1.01 |
| σ | 0.62959 | 0.45730 | 0.00205 | 0.00210 | 0.03502 | 0.30554 | 0.54920 | 0.84731 | 1.73881 | 1 |
| Penalized | deviance: | 32.7 |  |  |  |  |  |  |  |  |

Figure S: JAGS Code for Bayesian Binomial-Logit Random Effects Models

model{

#Likelihood

for (i in 1:Nstud){

r[i] ~ dbinom(p[i],n[i])

logit(p[i]) <- alpha[i]

alpha[i] ~ dnorm(mu.alpha,prec)

}

#Priors

mu.alpha ~ dnorm(0, 0.00001)

prec <- pow(sd,-2)

sd ~ dunif(0,10)

#Simulation

logit(pred.p) <- mu.alpha

}
