# Supplementary material for: The prevalence of occult leiomyosarcoma at surgery for presumed uterine fibroids: a meta-analysis
Source: Gynecol Surg. 2015 May 19;12(3):165–77. doi: 10.1007/s10397-015-0894-4 (PMC4532723; doi:10.1007/s10397-015-0894-4)
Supplement: Supplementary file 3 — Two tables detailing information regarding leiomyosarcomas uncovered by this search. Table 1 provides details of the studies in which leiomyosarcomas were found and the number found, presence or absence of histopathology, age, data collection period, whether or not morcellation occurred, and outcome. Table 2 provides detail of the reported histopathology of each leiomyosarcoma. (PPTX 70 kb) [file 10397_2015_894_MOESM3_ESM.pptx]

## Slide 1
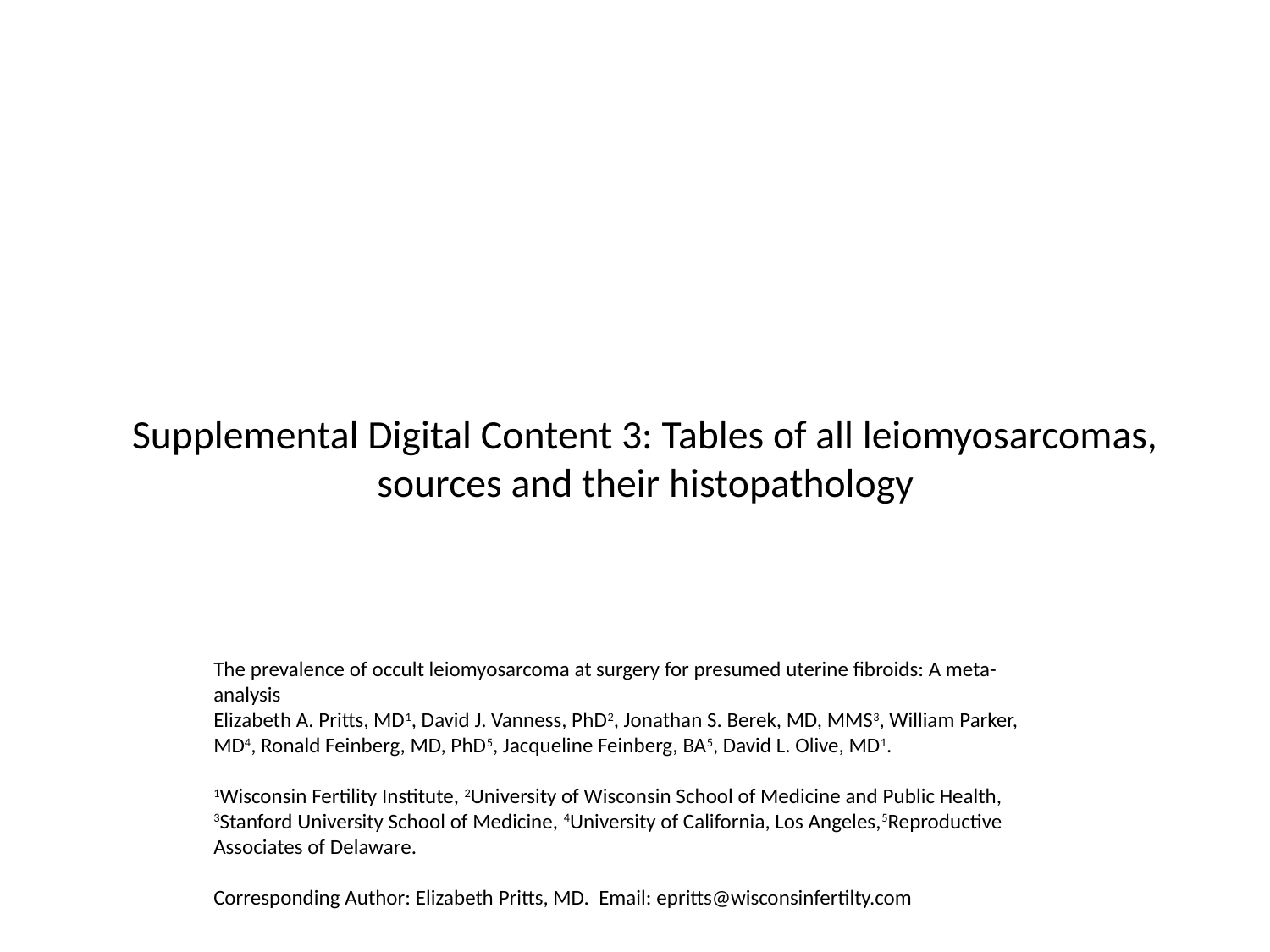

# Supplemental Digital Content 3: Tables of all leiomyosarcomas, sources and their histopathology
The prevalence of occult leiomyosarcoma at surgery for presumed uterine fibroids: A meta-analysis
Elizabeth A. Pritts, MD1, David J. Vanness, PhD2, Jonathan S. Berek, MD, MMS3, William Parker, MD4, Ronald Feinberg, MD, PhD5, Jacqueline Feinberg, BA5, David L. Olive, MD1.
 
1Wisconsin Fertility Institute, 2University of Wisconsin School of Medicine and Public Health, 3Stanford University School of Medicine, 4University of California, Los Angeles,5Reproductive Associates of Delaware.
 
Corresponding Author: Elizabeth Pritts, MD. Email: epritts@wisconsinfertilty.com

## Slide 2
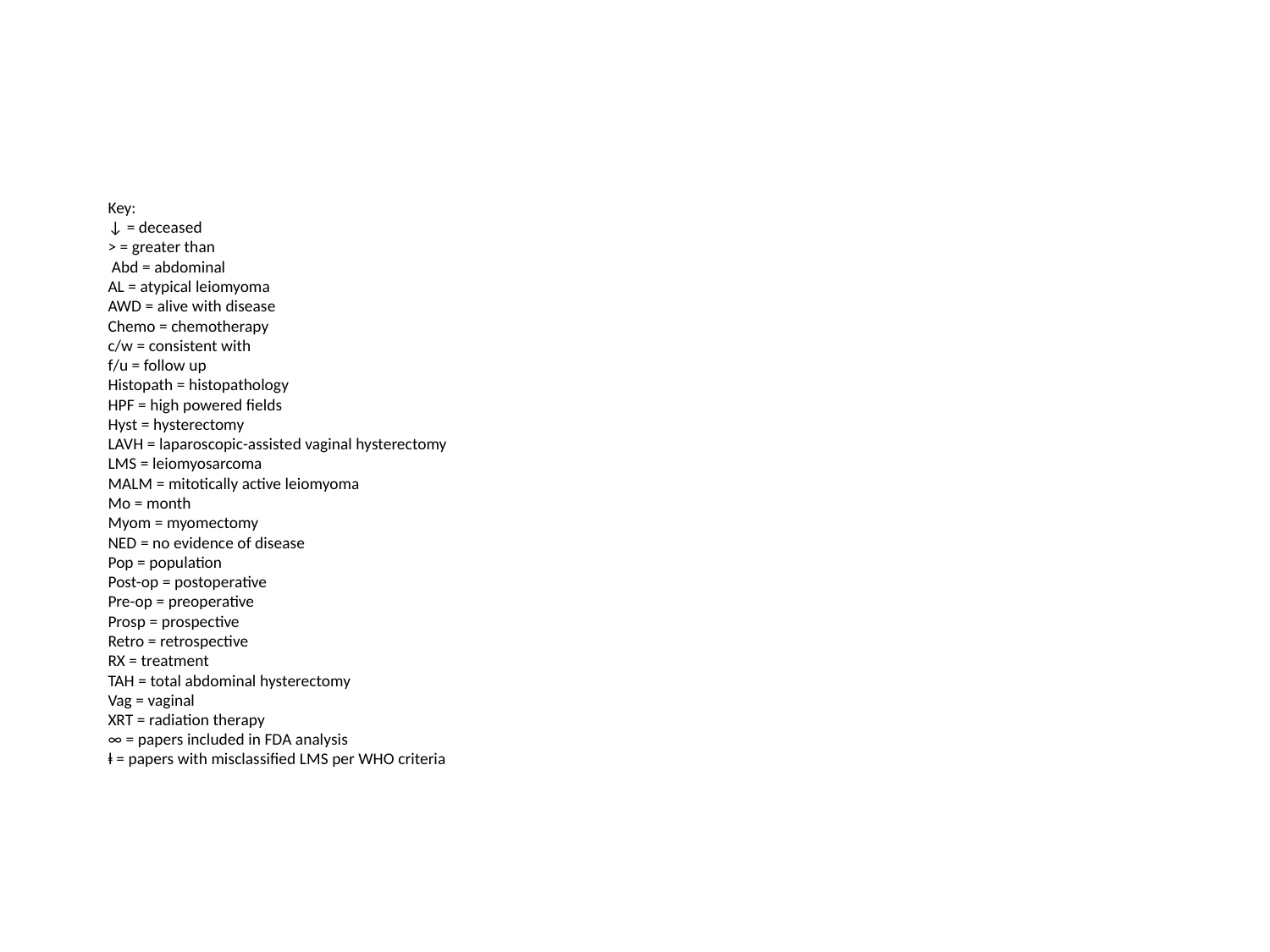

# Key: ↓ = deceased > = greater than Abd = abdominal AL = atypical leiomyoma AWD = alive with disease Chemo = chemotherapy c/w = consistent with f/u = follow up Histopath = histopathology HPF = high powered fields Hyst = hysterectomy LAVH = laparoscopic-assisted vaginal hysterectomy LMS = leiomyosarcoma MALM = mitotically active leiomyoma Mo = month Myom = myomectomy NED = no evidence of disease Pop = population Post-op = postoperative Pre-op = preoperative Prosp = prospective Retro = retrospective RX = treatment TAH = total abdominal hysterectomy Vag = vaginal XRT = radiation therapy ∞ = papers included in FDA analysis ⱡ = papers with misclassified LMS per WHO criteria

## Slide 3
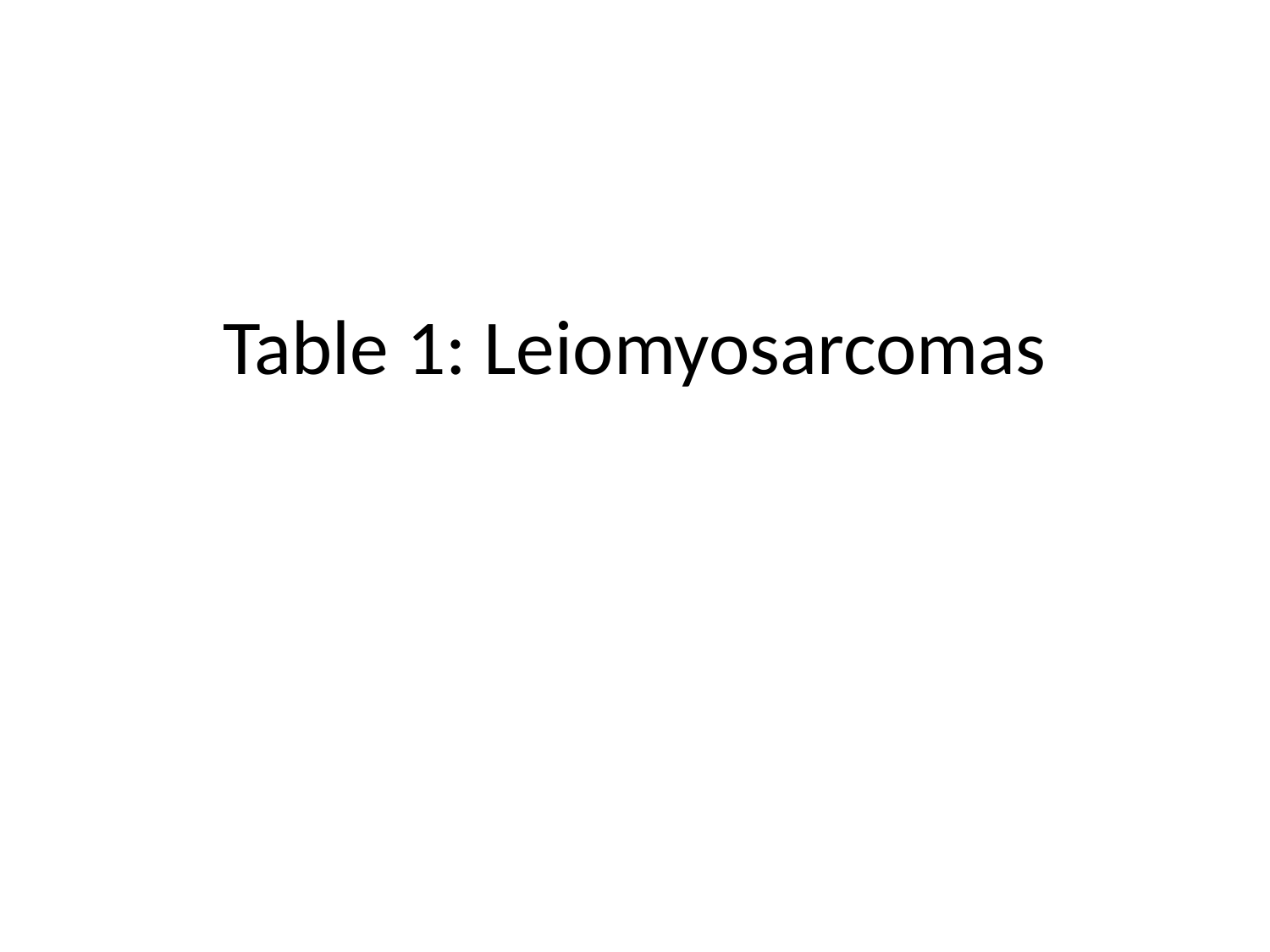

# Table 1: Leiomyosarcomas

## Slide 4
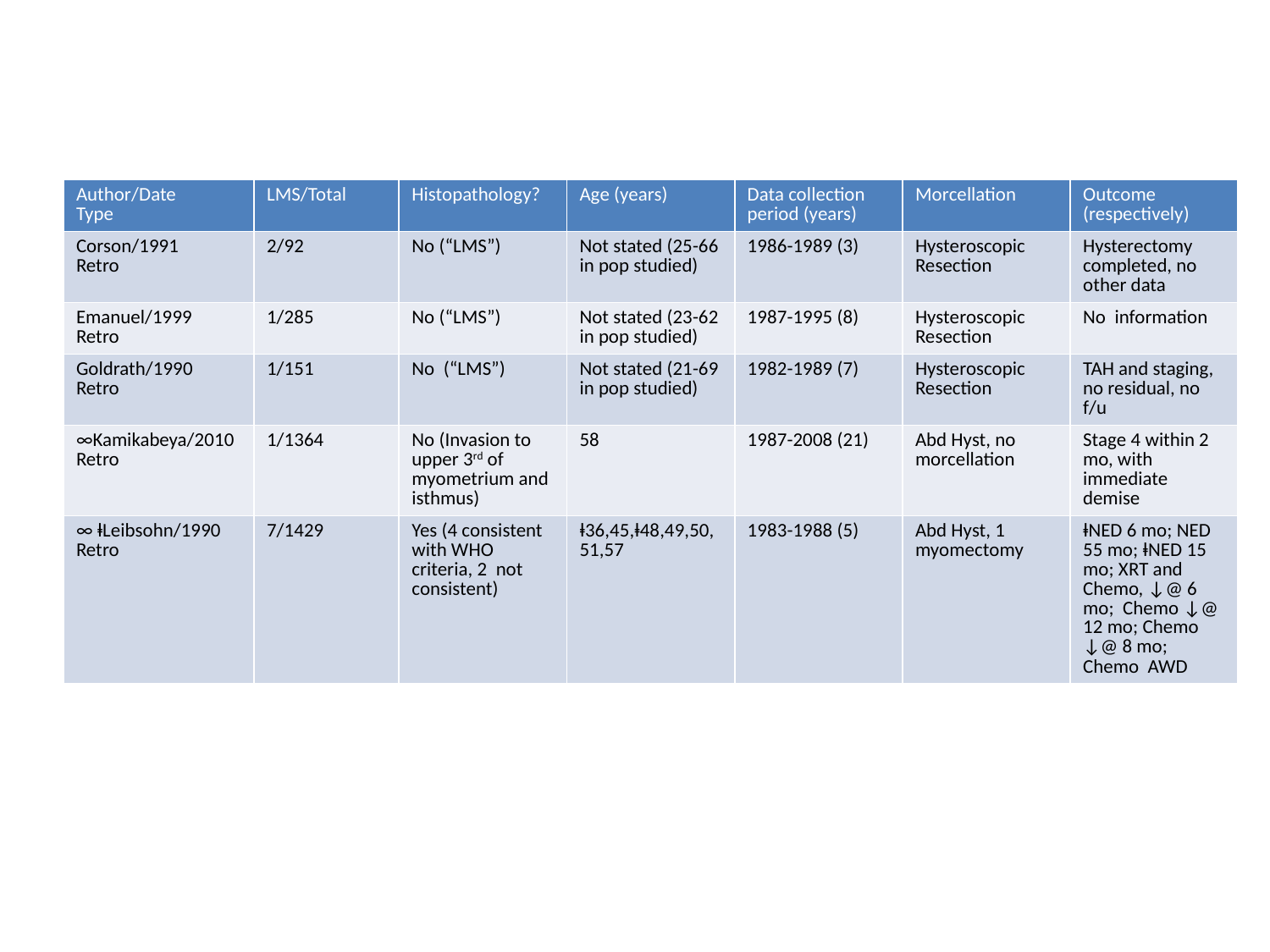

#
| Author/Date Type | LMS/Total | Histopathology? | Age (years) | Data collection period (years) | Morcellation | Outcome (respectively) |
| --- | --- | --- | --- | --- | --- | --- |
| Corson/1991 Retro | 2/92 | No (“LMS”) | Not stated (25-66 in pop studied) | 1986-1989 (3) | Hysteroscopic Resection | Hysterectomy completed, no other data |
| Emanuel/1999 Retro | 1/285 | No (“LMS”) | Not stated (23-62 in pop studied) | 1987-1995 (8) | Hysteroscopic Resection | No information |
| Goldrath/1990 Retro | 1/151 | No (“LMS”) | Not stated (21-69 in pop studied) | 1982-1989 (7) | Hysteroscopic Resection | TAH and staging, no residual, no f/u |
| ∞Kamikabeya/2010 Retro | 1/1364 | No (Invasion to upper 3rd of myometrium and isthmus) | 58 | 1987-2008 (21) | Abd Hyst, no morcellation | Stage 4 within 2 mo, with immediate demise |
| ∞ ⱡLeibsohn/1990 Retro | 7/1429 | Yes (4 consistent with WHO criteria, 2 not consistent) | ⱡ36,45,ⱡ48,49,50,51,57 | 1983-1988 (5) | Abd Hyst, 1 myomectomy | ⱡNED 6 mo; NED 55 mo; ⱡNED 15 mo; XRT and Chemo, ↓@ 6 mo; Chemo ↓@ 12 mo; Chemo ↓@ 8 mo; Chemo AWD |

## Slide 5
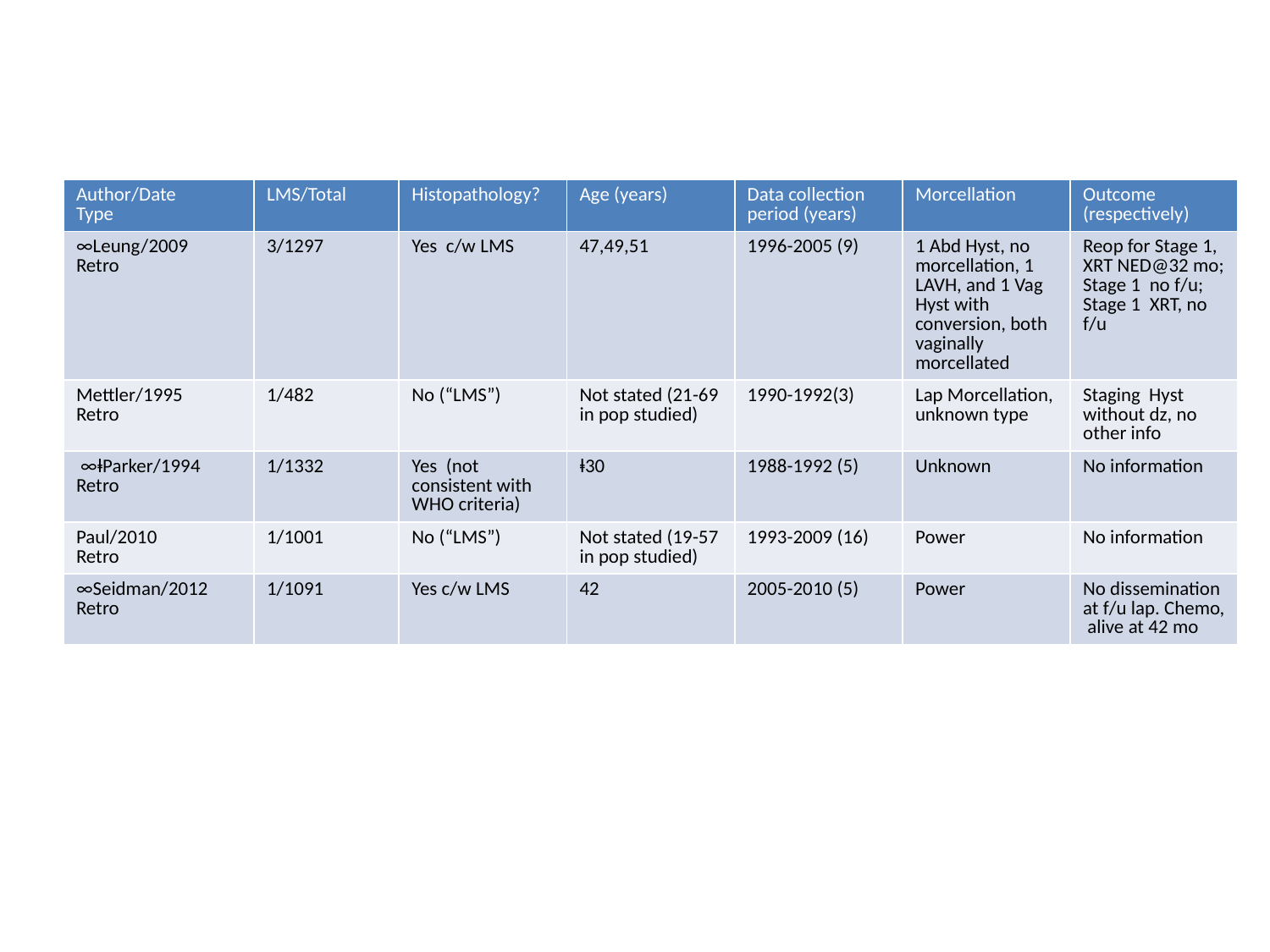

#
| Author/Date Type | LMS/Total | Histopathology? | Age (years) | Data collection period (years) | Morcellation | Outcome (respectively) |
| --- | --- | --- | --- | --- | --- | --- |
| ∞Leung/2009 Retro | 3/1297 | Yes c/w LMS | 47,49,51 | 1996-2005 (9) | 1 Abd Hyst, no morcellation, 1 LAVH, and 1 Vag Hyst with conversion, both vaginally morcellated | Reop for Stage 1, XRT NED@32 mo; Stage 1 no f/u; Stage 1 XRT, no f/u |
| Mettler/1995 Retro | 1/482 | No (“LMS”) | Not stated (21-69 in pop studied) | 1990-1992(3) | Lap Morcellation, unknown type | Staging Hyst without dz, no other info |
| ∞ⱡParker/1994 Retro | 1/1332 | Yes (not consistent with WHO criteria) | ⱡ30 | 1988-1992 (5) | Unknown | No information |
| Paul/2010 Retro | 1/1001 | No (“LMS”) | Not stated (19-57 in pop studied) | 1993-2009 (16) | Power | No information |
| ∞Seidman/2012 Retro | 1/1091 | Yes c/w LMS | 42 | 2005-2010 (5) | Power | No dissemination at f/u lap. Chemo, alive at 42 mo |

## Slide 6
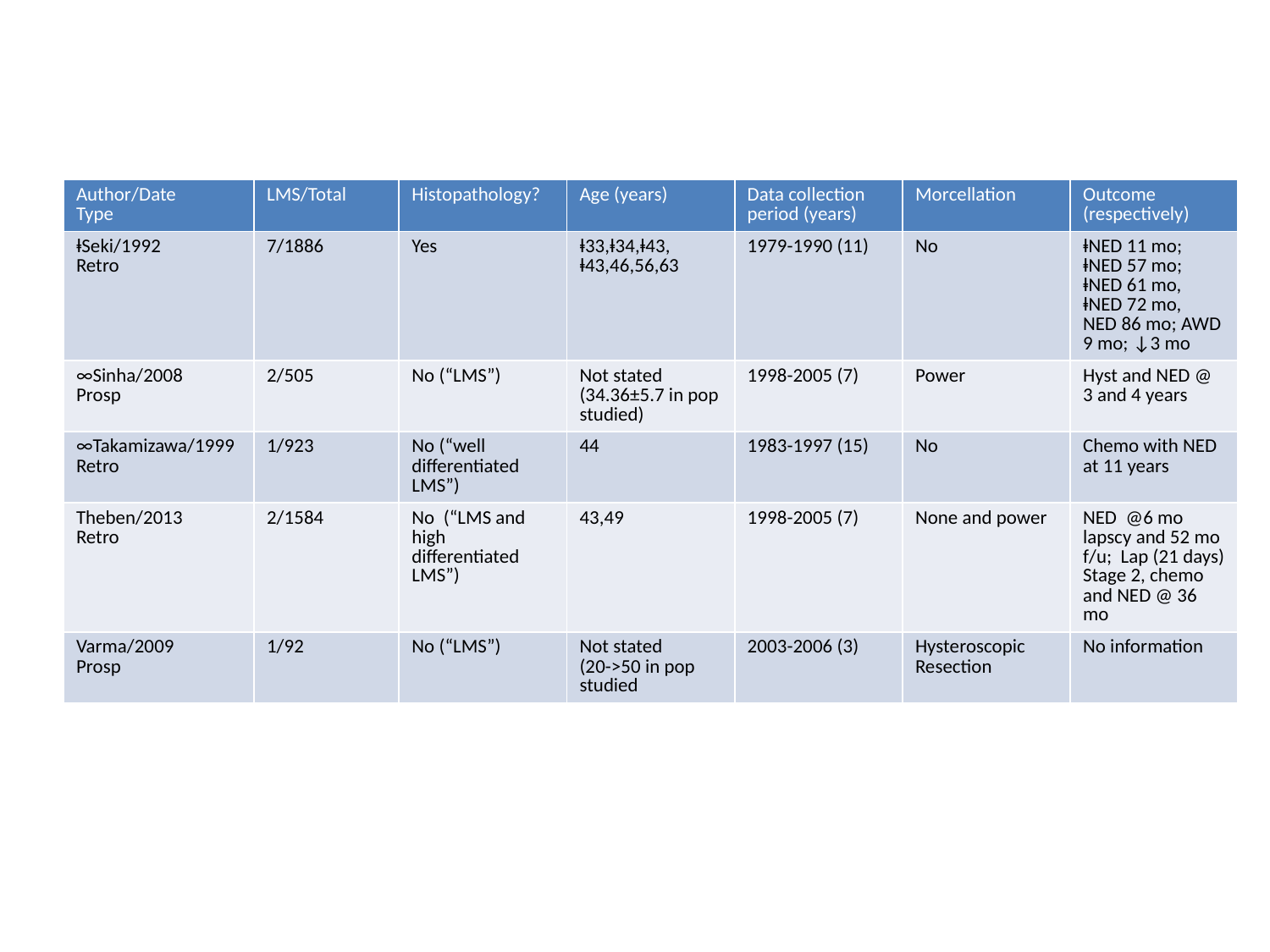

#
| Author/Date Type | LMS/Total | Histopathology? | Age (years) | Data collection period (years) | Morcellation | Outcome (respectively) |
| --- | --- | --- | --- | --- | --- | --- |
| ⱡSeki/1992 Retro | 7/1886 | Yes | ⱡ33,ⱡ34,ⱡ43, ⱡ43,46,56,63 | 1979-1990 (11) | No | ⱡNED 11 mo; ⱡNED 57 mo; ⱡNED 61 mo, ⱡNED 72 mo, NED 86 mo; AWD 9 mo; ↓3 mo |
| ∞Sinha/2008 Prosp | 2/505 | No (“LMS”) | Not stated (34.36±5.7 in pop studied) | 1998-2005 (7) | Power | Hyst and NED @ 3 and 4 years |
| ∞Takamizawa/1999 Retro | 1/923 | No (“well differentiated LMS”) | 44 | 1983-1997 (15) | No | Chemo with NED at 11 years |
| Theben/2013 Retro | 2/1584 | No (“LMS and high differentiated LMS”) | 43,49 | 1998-2005 (7) | None and power | NED @6 mo lapscy and 52 mo f/u; Lap (21 days) Stage 2, chemo and NED @ 36 mo |
| Varma/2009 Prosp | 1/92 | No (“LMS”) | Not stated (20->50 in pop studied | 2003-2006 (3) | Hysteroscopic Resection | No information |

## Slide 7
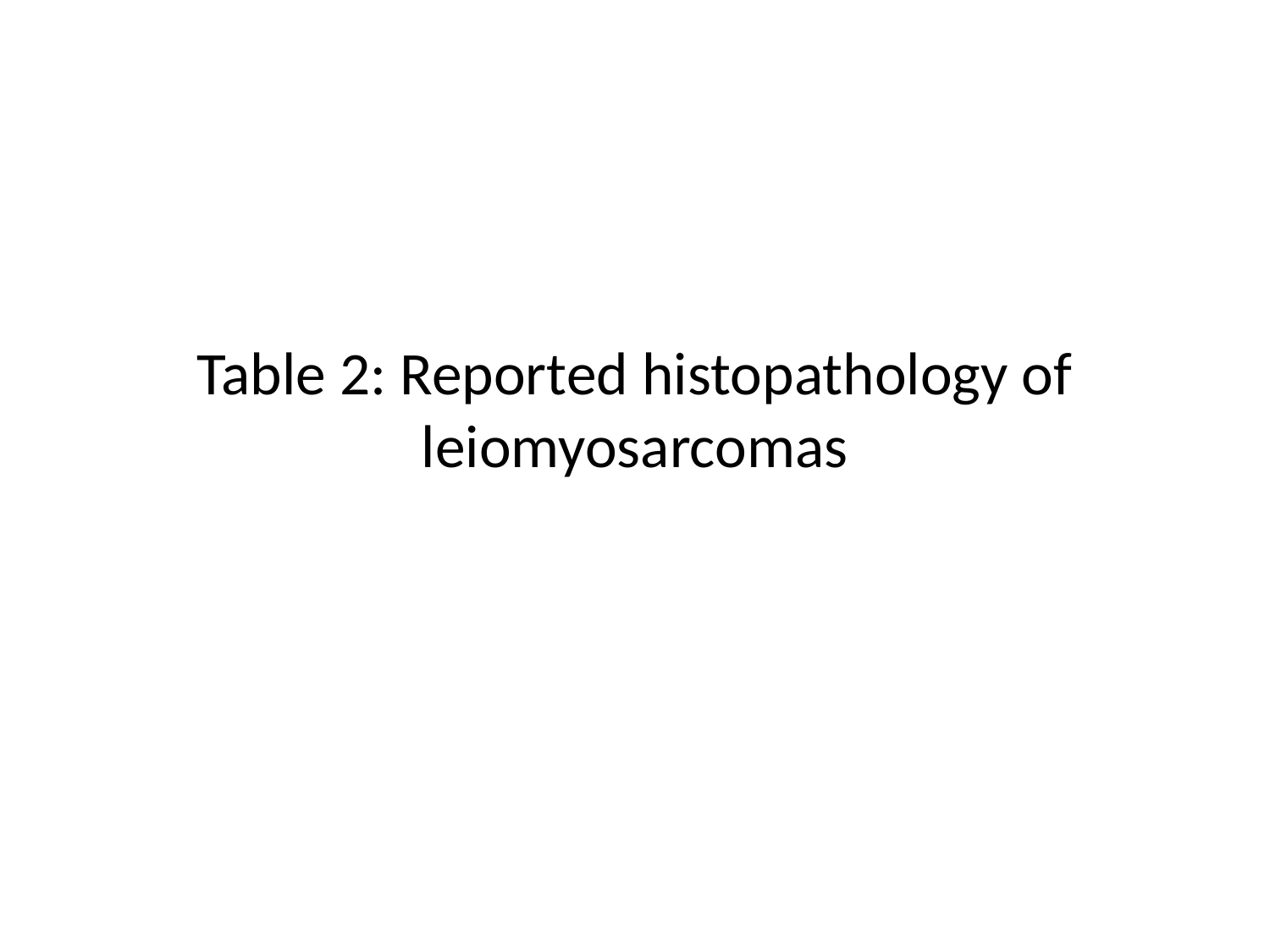

# Table 2: Reported histopathology of leiomyosarcomas

## Slide 8
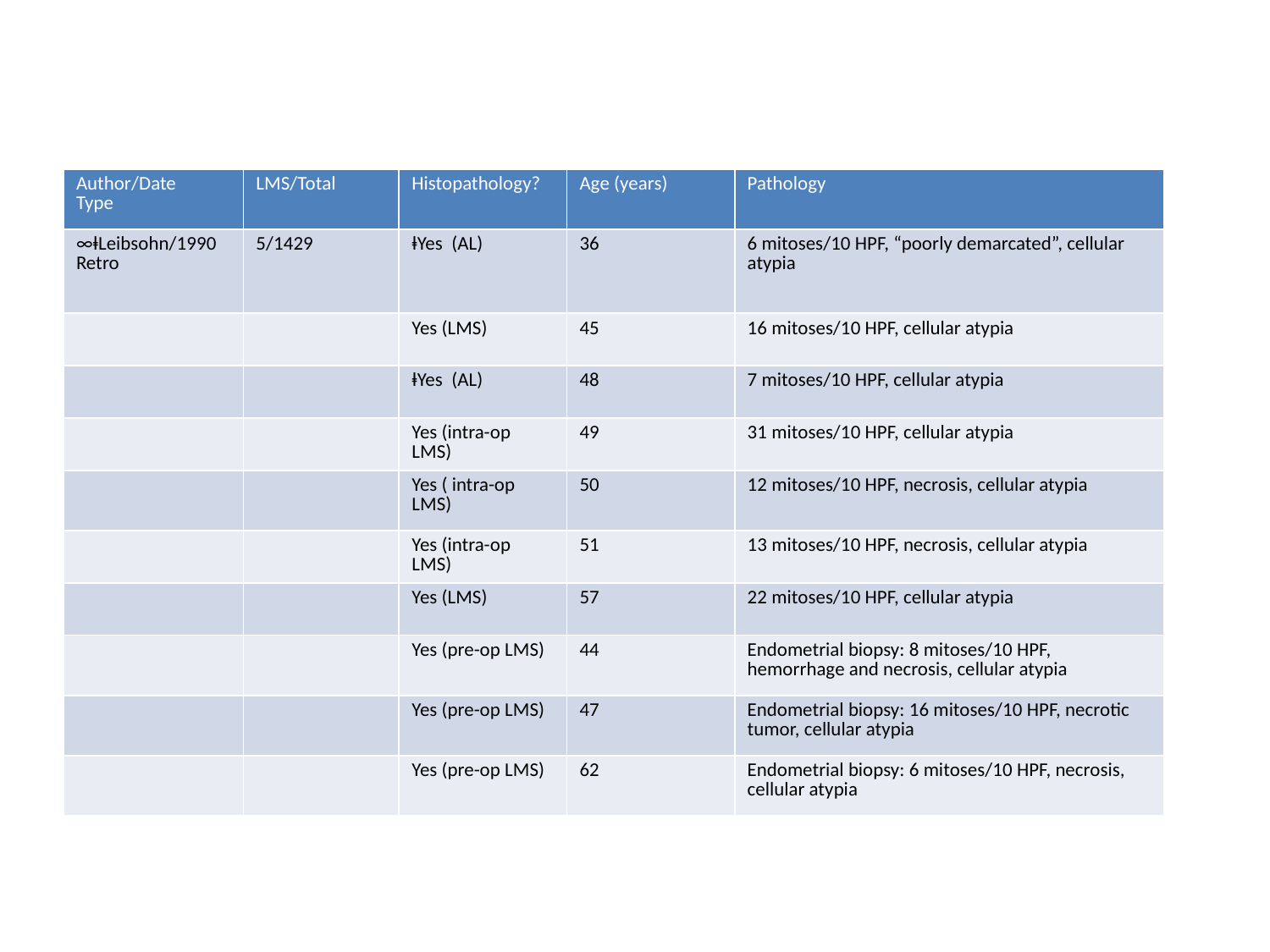

#
| Author/Date Type | LMS/Total | Histopathology? | Age (years) | Pathology |
| --- | --- | --- | --- | --- |
| ∞ⱡLeibsohn/1990 Retro | 5/1429 | ⱡYes (AL) | 36 | 6 mitoses/10 HPF, “poorly demarcated”, cellular atypia |
| | | Yes (LMS) | 45 | 16 mitoses/10 HPF, cellular atypia |
| | | ⱡYes (AL) | 48 | 7 mitoses/10 HPF, cellular atypia |
| | | Yes (intra-op LMS) | 49 | 31 mitoses/10 HPF, cellular atypia |
| | | Yes ( intra-op LMS) | 50 | 12 mitoses/10 HPF, necrosis, cellular atypia |
| | | Yes (intra-op LMS) | 51 | 13 mitoses/10 HPF, necrosis, cellular atypia |
| | | Yes (LMS) | 57 | 22 mitoses/10 HPF, cellular atypia |
| | | Yes (pre-op LMS) | 44 | Endometrial biopsy: 8 mitoses/10 HPF, hemorrhage and necrosis, cellular atypia |
| | | Yes (pre-op LMS) | 47 | Endometrial biopsy: 16 mitoses/10 HPF, necrotic tumor, cellular atypia |
| | | Yes (pre-op LMS) | 62 | Endometrial biopsy: 6 mitoses/10 HPF, necrosis, cellular atypia |

## Slide 9
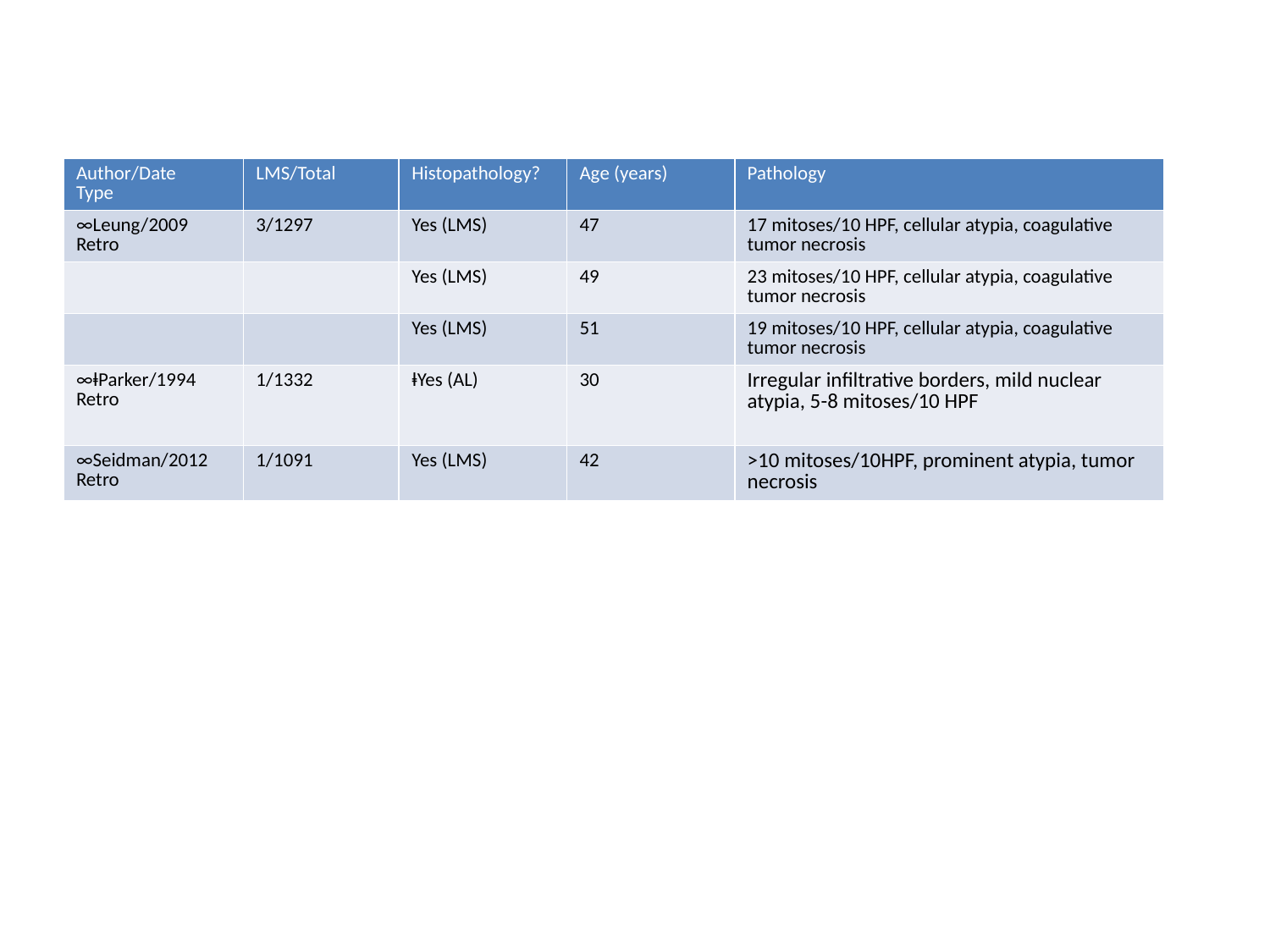

#
| Author/Date Type | LMS/Total | Histopathology? | Age (years) | Pathology |
| --- | --- | --- | --- | --- |
| ∞Leung/2009 Retro | 3/1297 | Yes (LMS) | 47 | 17 mitoses/10 HPF, cellular atypia, coagulative tumor necrosis |
| | | Yes (LMS) | 49 | 23 mitoses/10 HPF, cellular atypia, coagulative tumor necrosis |
| | | Yes (LMS) | 51 | 19 mitoses/10 HPF, cellular atypia, coagulative tumor necrosis |
| ∞ⱡParker/1994 Retro | 1/1332 | ⱡYes (AL) | 30 | Irregular infiltrative borders, mild nuclear atypia, 5-8 mitoses/10 HPF |
| ∞Seidman/2012 Retro | 1/1091 | Yes (LMS) | 42 | >10 mitoses/10HPF, prominent atypia, tumor necrosis |

## Slide 10
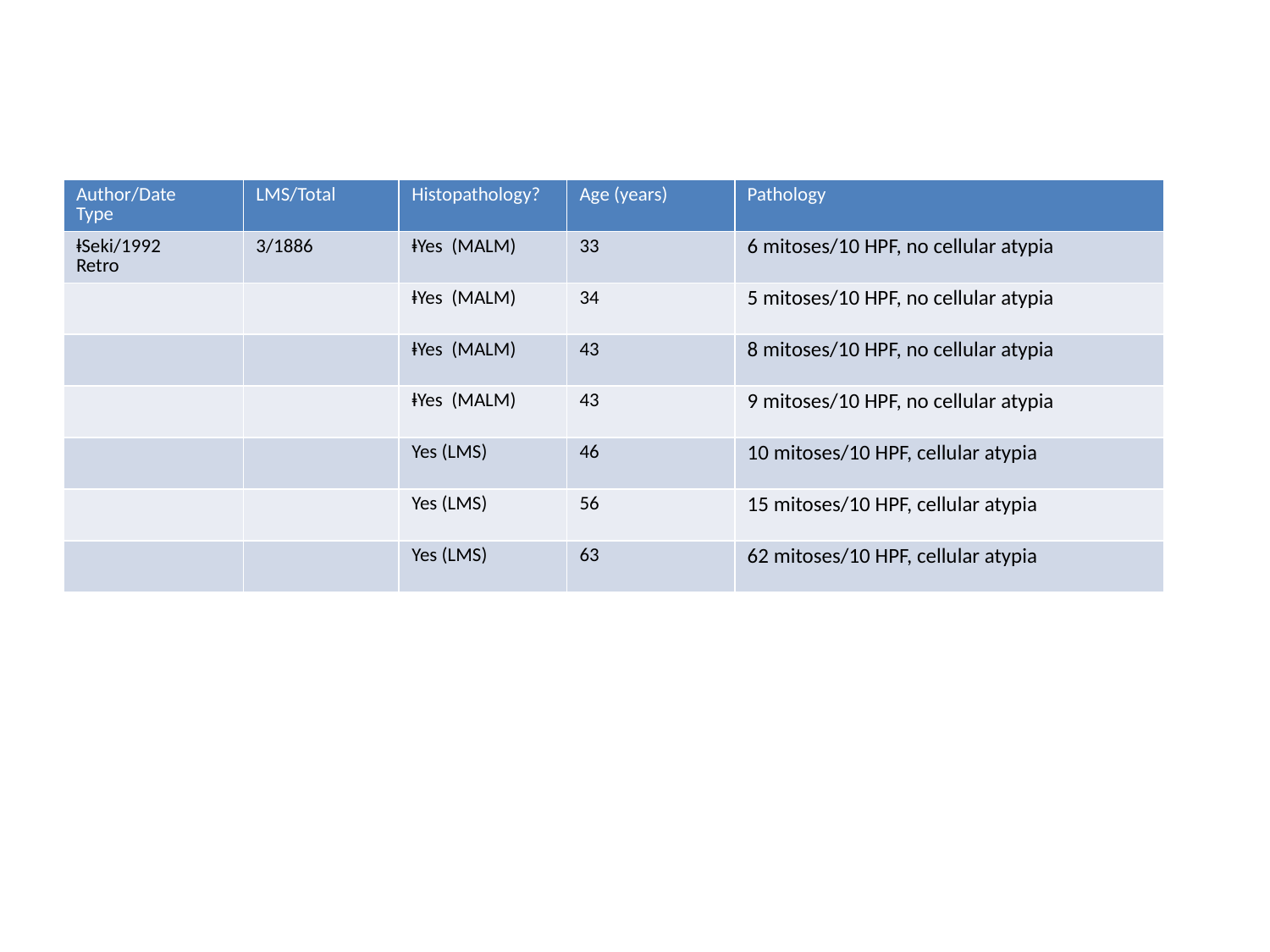

#
| Author/Date Type | LMS/Total | Histopathology? | Age (years) | Pathology |
| --- | --- | --- | --- | --- |
| ⱡSeki/1992 Retro | 3/1886 | ⱡYes (MALM) | 33 | 6 mitoses/10 HPF, no cellular atypia |
| | | ⱡYes (MALM) | 34 | 5 mitoses/10 HPF, no cellular atypia |
| | | ⱡYes (MALM) | 43 | 8 mitoses/10 HPF, no cellular atypia |
| | | ⱡYes (MALM) | 43 | 9 mitoses/10 HPF, no cellular atypia |
| | | Yes (LMS) | 46 | 10 mitoses/10 HPF, cellular atypia |
| | | Yes (LMS) | 56 | 15 mitoses/10 HPF, cellular atypia |
| | | Yes (LMS) | 63 | 62 mitoses/10 HPF, cellular atypia |
